# An amino acid-defined diet impairs tumour growth in mice by promoting endoplasmic reticulum stress and mTOR inhibition

**DOI:** 10.1016/j.molmet.2022.101478

**Published:** 2022-03-30

**Authors:** Maurizio Ragni, Chiara Ruocco, Laura Tedesco, Michele O. Carruba, Alessandra Valerio, Enzo Nisoli

**Affiliations:** 1 Center for Study and Research on Obesity, Department of Biomedical Technology and Translational Medicine, University of Milan, Via Vanvitelli 32, 20129, Milan, Italy; 2 Department of Molecular and Translational Medicine, Brescia University, Viale Europa 11, 25123, Brescia, Italy

**Keywords:** Branched-chain amino acids, Cancer metabolism, Essential amino acids, Glycolysis, Mechanistic/mammalian target of rapamycin, Mitochondria

## Abstract

**Objective:**

Profound metabolic alterations characterize cancer development and, beyond glucose addiction, amino acid (AA) dependency is now recognized as a hallmark of tumour growth. Therefore, targeting the metabolic addiction of tumours by reprogramming their substrate utilization is an attractive therapeutic strategy. We hypothesized that a dietary approach targeted to stimulate oxidative metabolism could reverse the metabolic inflexibility of tumours and represent a proper adjuvant therapy.

**Methods:**

We measured tumour development in xenografted mice fed with a designer, casein-deprived diet enriched in free essential amino acids (EAAs; SFA-EAA diet), or two control isocaloric, isolipidic, and isonitrogenous diets, identical to the SFA-EAA diet except for casein presence (SFA diet), or casein replacement by the free AA mixture designed on the AA profile of casein (SFA-CAA diet). Moreover, we investigated the metabolic, biochemical, and molecular effects of two mixtures that reproduce the AA composition of the SFA-EAA diet (*i.e.*, EAAm) and SFA-CAA diet (*i.e.*, CAAm) in diverse cancer and non-cancer cells.

**Results:**

The SFA-EAA diet reduced tumour growth *in vivo*, promoted endoplasmic reticulum (ER) stress, and inhibited mechanistic/mammalian target of rapamycin (mTOR) activity in the tumours. Accordingly, in culture, the EAAm, but not the CAAm, activated apoptotic cell death in cancer cells without affecting the survival and proliferation of non-cancer cells. The EAAm increased branched-chain amino acid (BCAA) oxidation and decreased glycolysis, ATP levels, redox potential, and intracellular content of selective non-essential amino acids (NEAA) in cancer cells. The EAAm-induced NEAA starvation activated the GCN2-ATF4 stress pathway, leading to ER stress, mTOR inactivation, and apoptosis in cancer cells, unlike non-cancer cells.

**Conclusion:**

Together, these results confirm the efficacy of specific EAA mixtures in promoting cancer cells’ death and suggest that manipulation of dietary EAA content and profile could be a valuable support to the standard chemotherapy for specific cancers.

## Highlights

• Manipulating the amino acid content of diet affects metabolic homeostasis.
• A designer diet enriched in essential amino acids blocks the tumour growth in transplanted mice.
• The essential amino acids activate the mitochondrial BCAA oxidation and ER stress, inhibiting glycolysis and mTOR signalling in tumour cells.
• Targeting cancer metabolic inflexibility with designer diets may represent a promising adjuvant therapeutic approach.

## Graphical abstract

**Figure:**
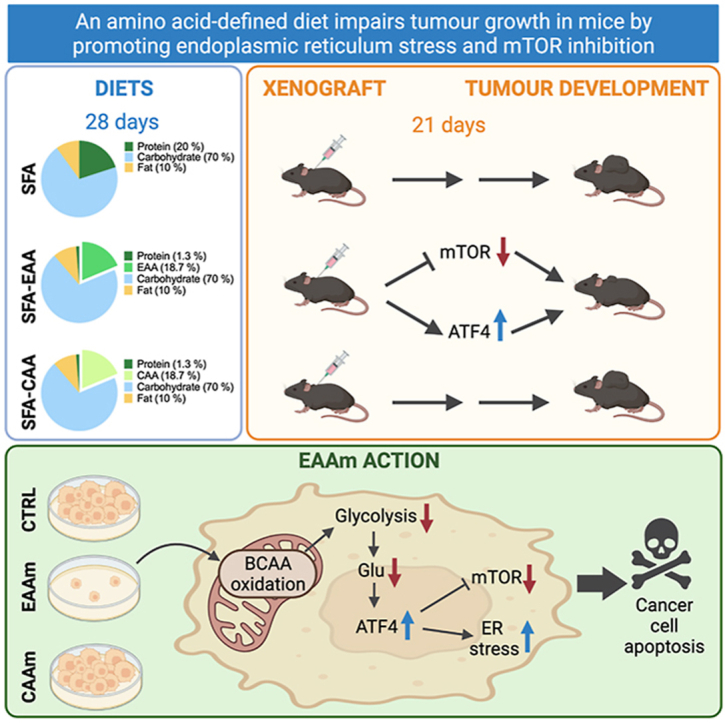

## 1 Introduction

Although cancer is a complex disease, alteration in its energy metabolism is the most significant hallmark [1,2]. Cancer cells display, in fact, extensive metabolic reprogramming toward glucose utilization, with almost exclusive reliance on aerobic glycolysis (*i.e*., the Warburg effect) [3]. This process is in contrast to those observed in normal, healthy cells, which can handle different substrates and switch on mitochondrial utilization of fat, glucose, or amino acids based on the different external inputs (*e.g*., dietary regimens, physical exercise, or daily circadian rhythms), or change their utilization in response to activity or rest, or fasting–feeding transition. Therefore, metabolic flexibility is a feature of healthy organisms, and dysregulation in the utilization of energetic substrates underlies, besides cancer, several other metabolic diseases, such as diabetes and obesity [[4], [5], [6]]. Furthermore, cancer development is affected by dietary habits, with an excess of animal protein, fat, and refined carbohydrate playing a relevant role, thus strongly supporting the concept of a disease influenced by the supply of metabolic substrates [7]. The unhealthy Western diet enriched in processed meats, sugar beverages, and excess salt is associated with a significantly increased risk of cancer [8].

On the other hand, dietary restriction of both fat and carbohydrate has been demonstrated to reduce cancer incidence and progression. Likewise, protein restriction and manipulation of the amino acid (AA) content, such as restriction of single AAs, prevent cancer development in mice [[10], [11], [12], [9]], thus suggesting a promising anti-cancer approach. Notably, most dietary manipulation strategies of these types exert a healthy metabolic outcome through improved metabolic flexibility and restoring appropriate energy substrate selection and utilization.

It is well-known that, beyond their role as building blocks for protein synthesis, AAs are involved in several other cellular functions, acting as both metabolic intermediates and fuel for biosynthetic reactions, redox homeostasis, epigenetic and post-transcriptional modifications [[13], [14], [15], [16]]. Therefore, balanced supply and proper management of AA metabolism are crucial for cell function and survival. Also, cancer cells display strong AA dependency through glutamine addiction, which significantly supports the Warburg effect [17,18]

Given the crucial roles of AAs in cell physiology and disease, cells have evolved two main AA-sensing pathways to cope with AA sufficiency, the mammalian target of rapamycin (mTOR) and general control nonderepressible 2 kinase (GCN2) [19]. mTOR comprises two protein complexes, mTORC1 and mTORC2 (mTOR). In particular, the AA-activated mTORC1, through downstream effectors, such as p70S6 kinase (p70S6K) and S6 ribosomal protein (S6), is the primary sensor that links nutrient availability to protein synthesis and cell proliferation [20,21]. AA starvation, in turn, activates GCN2 that, by sensing uncharged tRNA levels [22], triggers the integrated stress response and, through the activating transcription factor 4 (ATF4), leads to the AA-starvation response as part of the endoplasmic reticulum (ER) stress process [19,22,23]. Both mTORC1 and GCN2-ATF4 signalling pathways are thus critically involved in the cell cycle, cell differentiation, and cell death [[24], [25], [26]], and their dysregulation is linked to multiple diseases, including cancer [19,27].

As rapidly growing cells and in continuous need of biomass, cancers are particularly susceptible to alterations in AA levels and their homeostatic mechanisms [10,28,29]; this addiction, therefore, pinpoints a metabolic vulnerability and a potential therapeutic target, which can be exploited by manipulating dietary macronutrients.

We have previously investigated the beneficial effects of amino acid supplementation in drinking water with both specific branched-chain amino acid (BCAA)-enriched mixtures (BCAAem) and AA mixtures added with tricarboxylic acid cycle (TCA) cycle intermediates (α5) in diverse pathological contexts. Indeed, BCAAem prevents sarcopenia and cognitive dysfunction during ageing [30,31] and steatosis in an experimental model of alcoholic liver disease [32], mitigates the dystrophic phenotype in the experimental model of Duchenne muscular dystrophy [33] and preserves organ damage induced by rosuvastatin [34], while α5 inhibits the Doxorubicin-induced mitochondrial dysfunction and oxidative stress [35]

More recently, we demonstrated that feeding mice with a designer diet, in which protein component (*i.e*., casein) was substituted with a defined AA formula enriched in EAAs (SFA-EAA diet), extended healthspan by preventing and reversing fat accumulation in obesity mouse models with beneficial effects on whole energy metabolism [36]. Notably, the favourable effects of the SFA-EAA diet on body weight, glucose homeostasis, and brown adipose tissue thermogenesis were accompanied by profound metabolic changes, characterized by increased fat oxidation, amelioration of impaired glucose disposal, and increased mitochondrial oxygen consumption, which restored the metabolic derangements induced by both dietary and genetic obesity. Since pharmacological reversing of the metabolic addiction and inflexibility which underlies oncogenic transformation has long been proved as a valuable anti-cancer strategy [[37], [38], [39]], we tested the ability of the substituted SFA-EAA diet to inhibit cancer development in mice. Here, we demonstrate that our original diet prevented tumour growth by inhibiting mTOR activity and stimulating ER stress. Both effects were linked to selective death of cancer cells through apoptosis, accompanied by inhibition of glycolysis and ATP production. Our findings underline the potential beneficial effects of specific dietary strategies, including an AA-substituted diet, to prevent tumour development in mammals or to integrate the tumour standard therapy in humans.

## 2 Material and methods

### 2.1 Animals, xenografts, and diets

For xenografts experiments, thirty male C57BL/6 N mice (8 weeks old) from Charles River (Calco, Italy) were weight-matched and fed *ad libitum* with saturated fatty acids (SFA) diet (20% protein − namely casein −, 70% carbohydrate, and 10% fat, half of which was lard (D12450H), or two isocaloric, isolipidic, and isonitrogenous diets, identical to SFA diet except for protein content (casein) which was almost entirely (93.5%) replaced with a defined formula of free EAAs (SFA-EAA, D14032501) or with an amino acid formula designed on the amino acid profile of casein (SFA-CAA diet, A17092801) all from Research Diets Inc. (Brogaarden, Gentofte, Denmark) (Supplementary Table 1) [36]. Bodyweight and food intake were recorded twice a week in mice housed individually. After one week, B16F10 melanoma cells (2 × 10^4^ cells) were injected into the flank of mice. Tumour development was monitored for the subsequent two weeks, after which mice were killed, and the tumour was explanted, snap-frozen, and stored at −80 °C until analysis. Tumour mass was measured daily with a caliper, and its volume was determined as V = (W^2^ × L)/2 where V is tumour volume, W is tumour width, and L is tumour length [40]. Animals were maintained under standard laboratory conditions (12 h light/dark cycle) with *ad libitum* access to water and food. All the animal procedures were conducted under the European Community Guidelines (Directive 2010/63/EU for animal experiments), and the Italian Ministry of Health, complied with the National Animal Protection Guidelines and approved by the Institutional Animal Ethical Committee.

### 2.2 Cell cultures

Cell lines used were: B16F10 (mouse melanoma, ATCC # CRL-6475), C2C12 (mouse myoblasts, ATCC # CRL-1772), HeLa (Human adenocarcinoma, ATCC # CCL-2), Detroit 573 (human fibroblasts, ATCC # CCL-117), NIH3T3 (mouse embryonic fibroblasts, ATCC # CRL-1658), HepG2 (human hepatocellular carcinoma, ATCC # HB-8065), SH-SY5Y (human neuroblastoma, ATCC # CRL-2266), M14 (human melanoma), HCT116 (human colorectal carcinoma) and HCT116 TP53^(−/−)^ (p53 null HCT116 cells) (a gift from Prof. Vincenzo Flati), HL-1 (mouse cardiomyocytes, Sigma Aldrich - SCC065). Cells were routinely grown in Dulbecco's modified Eagle's medium (DMEM), except for Hepg2, SH-SY5Y and HL-1, cultured in RPMI 1640, and Claycomb Medium (HL-1). All cell lines were used at a passage number from 5 to 10. Media were supplemented with 10% fetal bovine serum, 1% penicillin/streptomycin, 1% l-glutamine, and 1% Na^+^-pyruvate and were maintained at 37 °C, 5% CO_2_. All supplements and serum were from Euroclone (Milan, Italy). For *in vitro* experiments, cells were supplemented with culture media only, an EAAm mixture [30], or a mixture reproducing the amino acid composition of casein of rodent diets (see text for explanation) (both mixtures at 1% w/v). AAs were dissolved in the appropriate culture medium for the indicated period. Plasma was obtained from xenografted animals fed with the three experimental diets (section 2.12) and was supplemented to culture media in serial dilutions; cell viability was assessed by the MTT method (see below). For culture in galactose, M14 cells were grown in DMEM without glucose supplemented with 25 mM galactose (Sigma Aldrich) (M14gal), in parallel with M14 cultures grown in standard DMEM high glucose (M14). M14gal cells were then allowed to grow and adapt to galactose for several passages. Afterwards, aliquots were frozen and used for later experiments, together with the same passage number batches of M14. 3,6-Dichloro-1-benzothiophene-2-carboxylic acid (BT2) (Santa Cruz Biotechnology # sc-276559) and Rapamycin (ENZO life sciences # BML-A275) were dissolved in dimethyl-sulfoxide (DMSO) as stock solutions and diluted in culture media at the indicated concentrations.

### 2.3 Viability assay

The viability of cells was determined using the standard MTT [3-(4,5-dimethylthiazol-2-yl)-2,5-diphenyltetrazolium bromide] assay. All the treatments were done using 8 × 10^3^ to 2 × 10^4^ cells/well, depending on the cell line used, in 96 wells plate in 100 μL of the medium. The purple formazan crystals were dissolved overnight at 37 °C in 5% SDS/0.1 M HCl (100 μL/well), and the absorbance was recorded on a microplate reader at a dual-wavelength of 570 nm/655 nm. Alternatively, cell number was determined using Sulforhodamine B based *in vitro* Toxicology Assay Kit (Sigma TOX6), following the manufacturer's instruction.

### 2.4 Wound healing assay

Cells were grown to full confluence in 24-well plates in their respective growth media and were then incubated overnight in control or EAAm media. Cell cultures were then scratched with a 200 μL sterile pipette tip and extensively washed with PBS to remove detached cells and debris. The microscopy images were collected after 16 h to assess migration ability recovery.

### 2.5 Clonogenic survival assay

One hundred to 1000 cells were seeded in triplicate in six-well plates one day before treatment with a control DMEM or EAAm mixture. Cells were then allowed to grow in DMEM or EAAm media until they formed colonies for about ten days. Survived colonies were fixed with 100% methanol and stained with methylene blue in methanol. Experiments were repeated at least three times with three biological replicates.

### 2.6 Quantitative real-time polymerase chain reaction (qRT-PCR)

RNA was isolated from cells using the RNA easy Mini Kit (Qiagen, Milan, Italy), and cDNA was synthesized using iScript cDNA Synthesis Kit (Bio-Rad Laboratories, Segrate, Italy). The mRNA levels were measured by quantitative Real-Time quantitative PCR in triplicate, with iTaq Universal SYBR Green SuperMix (Bio-Rad Laboratories) on a CFX Connect Real-Time PCR System (Bio-Rad Laboratories). Primers were designed using Primer3 (version 0.4.0) software. The cycle number at which the various transcripts were detectable (threshold cycle, CT) was compared with CT values of glyceraldehyde-3-phosphate-dehydrogenase (GAPDH), which was used as the housekeeping gene, and was referred to as ΔCT. The relative gene level was expressed as 2^−(ΔΔCT),^ where ΔΔCT corresponded to the difference between the ΔCT of either treatment group and the ΔCT of the control group.

Mouse primers used were: CHOP (Forward: 5′-ACGGAAACAGAGTGGTCAGT-3′, Reverse 5′AGACAGACAGGAGGTGATGC-3′), GRP78(Forward:5′-CCAGCGACAAGCAACCAAAG-3′, Reverse5′-GCCACCCAGGTCAAACACAA-3′), Chac1 (Forward:5′- TACCAAGTTCGAGGGGAGC-3′, Reverse 5′-TCTGTGTGGCAATGACCTCT-3′), Sestrin2(Forward:5′-GCCCCTGAGAAGCTCCGCAA-3′, Reverse5′-GAGTTCGGCCAGGGACCAGC-3′). Human primers sequences were: CHOP (Foward:5′-GCCTGGTATGAGGACCTGCAAGA-3′, Reverse5′-CTCCTCAGTCAGCCAAGCCAGAG-3′), GRP78(Forward:5′CTATTGGGGTGTTTCGCGAG-3′, Reverse5′-GAGAGCTTCATCTTGCCAGC-3′), Chac1 (Forward:5′CCATCGGGGCAGCGACAAGA-3′, Reverse5′-GGCCTTGCTTACCTGCTCCCC-3′), Sestrin-2 (Forward:5′-CTGCTGCGGGATGAGGGGAC-3′, Reverse5′-TGTCTGGGTGTGGAGAGGGCT-3′).

### 2.7 Small-interfering RNAs (siRNA) silencing

Cells were transfected with 50 nmol/l of siRNA using the 4D-Nucleofector system (Lonza), following optimized protocols for the cell line under examination. Predesigned siRNA (Dharmacon) was a pool of 4 non-targeting scrambled controls (# D-001810-10-05) or a pool of 4 specific siRNA against human Sestrin2 (# L-019134-02-0005) or human BCKDK (# L-004932-00-0005). After 24 h from transfection, cells were treated as described in figure legends.

### 2.8 Protein extraction and immunoblot analysis

Protein extracts were obtained using T-PER or M-PER Mammalian Protein Extraction Reagent (Pierce; ThermoScientific) as indicated by the manufacturer, in the presence of protease and phosphatase inhibitor cocktail (Sigma). For ATF4 protein expression measurement, western blots were performed on nuclear extracts (NE) obtained with the REAP method [40]. Protein content was determined with bicinchoninic acid (BCA) protein assays (Pierce). An appropriate amount (30–40 μg) of protein was run on 4–20% gradient TGX sodium dodecyl sulfate-polyacrylamide gel electrophoresis (SDS-PAGE) gels (BioRad). After the run, gels were transferred to nitrocellulose or PVDF, blocked and incubated with the specific antibodies. De novo protein synthesis measurement was performed with the SUnSET method [41]. Briefly, cells were pulsed with puromycin (1 μg/ml; Sigma–Aldrich) for 10 min, and, after incubation, total proteins were extracted as reported above. Puromycin incorporation was detected by immunoblotting with an anti-puromycin antibody (Millipore cat # MABE343). Antibodies used were: ATF4 (Santa Cruz Biotechnology cat # sc-200), p-70S6K (Thr389) (Cell Signaling cat # 9205), p70S6K (Cell Signaling cat # 9202), Phospho-S6 Ribosomal Protein (Ser235/236) (Cell Signaling cat # 4858) S6 Ribosomal Protein (Cell Signaling cat # 2217) Menin (Active Motifs cat # 2615023), CytC (Cell Signaling cat # 4280), COXIV (Cell Signaling cat # 4844), PARP (Trevigen # 4338), Caspase 3 (Cell Signaling cat # 9662), HIF-1α (BD Biosciences cat # 610958), β-Actin (Cell Signaling cat # 4970), p-eIF2α (Ser51) (Cell Signaling cat # 3398), eIF2α (Cell Signaling cat # 5324), PP2CM (Abcam cat # 135286), p-BCKDHA (Ser293) (Abcam cat # 200577), BCKDHA (Abcam cat # 138460), GAPDH (Cell Signaling cat # 2118), SESN-2 (Santa Cruz Biotechnology # sc-101249), BCKDK (Santa Cruz Biotechnology # sc-374425).

### 2.9 ATP amount, NADP^+^/NADPH ratio, and glucose uptake measurement

Adenosine triphosphate (ATP) and the ratio of oxidized/reduced nicotinamide-adenine-dinucleotide phosphate (NADP^+^/NADPH) levels were measured by colourimetric methods using commercially available kits (BioVision K354-100 for ATP and K347-100 for NADP^+^/NADPH ratio). Results were normalized to total protein content. Glucose uptake was assessed by measuring glucose depletion in a cell culture medium with a glucose-oxidase method (GO Assay Kit – Sigma Aldrich GAGO20). Glucose was measured in both control and supplemented cells at time zero (T_0_) and after 5 h of treatment (T_1_). The difference in absorbance (T_1_-T_0_)/min was assumed to be the glucose consumption rate and normalized vs cell number.

### 2.10 Oxygen consumption and glycolysis measurement

The rate of change of dissolved O_2_ (oxygen consumption rate, OCR) and glycolysis (as extracellular acidification rate, ECAR) was measured in the XF24 Analyzer (Seahorse Biosciences) following the manufacturer's instructions. Cells (2–4x10^3^ per well) were plated in XF24 cell culture microplates (Seahorse Biosciences, MA, USA) and, after 18 h, were equilibrated with DMEM lacking bicarbonate (Seahorse Biosciences) supplemented with glucose at 37 °C for 1 h in an incubator lacking CO_2_. For cells treatment, the EAAm mixture was dissolved in the equilibration medium and cells were treated for 1 h. For the mitochondrial stress test, after basal OCR measurement, oligomycin (2 μM), Carbonyl cyanide-4-(trifluoromethoxy)-phenylhydrazone (FCCP) (1 μM) and rotenone and antimycin A (both 0.5 μM) were then injected into the wells to monitor uncoupled, maximal and non-mitochondrial respiration, respectively.

### 2.11 NMR analysis

*Sample preparation*: NMR cell lysate samples were prepared into 5.00 mm NMR tubes (Bruker Bio Spin s.r.l) after the addition of 55 μL of 2H_2_O containing 10 mM sodium 3-trimethylsilyl [2,2,3,3-2H4] propionate (TMSP) to 495 μL of cell lysate.

*NMR experiments*: spectral acquisition and processing were performed according to standard procedures. 1H-NMR spectra were recorded with a Bruker 600 MHz (Bruker BioSpin) operating at 600.13 MHz proton Larmor frequency and equipped with a 5 mm PATXI 1H-13C-15 N and 2H-decoupling probe including a z-axis gradient coil, an automatic tuning-matching (ATM) and an automatic and refrigerate sample changer (SampleJet, Bruker BioSpin). A BTO 2000 thermocouple was served for temperature stabilization at approximately 0.1 K in the sample. Before measurement, samples were kept for 5 min inside the NMR probe head for temperature equilibration at 300 K.

For each sample, a monodimensional ^1^H NMR spectrum was acquired with water peak suppression and a standard NOESY pulse sequence using 128 scans, 65,536 data points, a spectral width of 12,019 Hz, an acquisition time of 2.7 s, a relaxation delay of 4 s and a mixing time of 0.01 s. The raw data were multiplied by a 0.3 Hz exponential line broadening before applying Fourier transform. Transformed spectra were automatically corrected for phase and baseline distortions and calibrated (chemical shift was referenced to the proton of TMSP at δ 0.0 ppm) using TopSpin 3.5 (Bruker Biospin Srl).

*Metabolite statistical analysis*: the multivariate and univariate analyses were performed using R 3.0.2 in house scripts. Unsupervised principal component analysis (PCA) was used to overview the data (visualization in a reduced space, clusters detection, screening for out-layers). Amino acids were assigned and their levels analyzed. The assignment procedure was made up using an NMR spectra library of pure organic compounds, public databases (*i.e*., Human Metabolome Database-HMBD), stored reference NMR spectra of metabolites, spiking NMR experiments and using data available in the literature. Matching between new NMR data and databases was performed using the AMIX software. The relative concentrations of the various metabolites were calculated by integrating the corresponding signals in the spectra with an in-house script.

### 2.12 Plasma and tumour free amino acid content

Blood was collected at the end of treatment by submandibular venipuncture (n = 3–4 mice/group). To obtained plasma, blood was collected into EDTA tubes (3 mM, Sigma–Aldrich, Milan, Italy) and centrifuged at 8000×*g* at 4 °C for 10 min. Free amino acid levels in plasma and tumour were measured by cation-exchange chromatography and detected spectrophotometrically after post-column reaction with ninhydrin reagent on a Biochrom 30+ Amino Acid Analyzer (Biochrom Ltd, ERRECI S.r.l Pieve Emanuele (MI) Italy) (n = 3–4 samples/group). Briefly, tumour samples were weighed, homogenized in 0.1% Triton X-100 loading buffer (250 μl/sample, Sigma Aldrich), and cold sonicated 30 s. Both plasma and tumour homogenate samples were mixed 1:1 in a 5-sulphosalicylic acid 5% (SSA)-based deproteinization reagent containing l-Norleucine (Sigma Aldrich), as internal standard (Cf = 250 μM). After an incubation phase (30 min at 4 °C), the samples were spin (l0000×*g* for 5 min 4 °C), and the supernatants were collected and filtered in column filters (0.22 μm; Millipore) (l2000×*g* for 10 min 4 °C). The calibration standard (Cf of each amino acid = 250 μM) (Biochrom Ltd) solution was treated with l-Norleucine SSA 5%-based buffer, in the same way as the samples. The standard solution and the samples, kept cold during the experiment, were injected into the analyzer (60 μl/sample). The amino acids levels were detected spectrophotometrically (440 and 570 nm) after post-column reaction with ninhydrin, under control reaction conditions at 135 °C. The absorbance provides a quantitative colorimetric assay for each amino acid.

### 2.13 Statistical analysis

Data were expressed as the mean ± standard error of the mean (SEM). Statistical analyses were performed using unpaired Student's t-test for two-group analysis or one-way or two-way ANOVA followed by post-hoc Tukey's test. For NMR metabolomic analysis, the non-parametric Wilcoxon test was used to determine the meaningful metabolites. Data were considered significant with p < 0.05. All statistical analyses were performed with GraphPad Prism (version 6.04).

## 3 Results

### 3.1 SFA-EAA diet blocks cancer growth *in vivo*

Supplementation of a specific EAA mixture in drinking water extended the average life span in old mice [30]. Furthermore, feeding an amino acid-defined diet prevented obesity development, reduced body weight gain in rodent genetic and dietary models, and prolonged healthy life span in mice [36]. Both dietary supplementation or manipulation exerted their metabolic effects by increasing mitochondrial activity and substrate oxidation; in particular, the designer diet induced a switch toward fat oxidation and improved the obesity-induced derangement in glucose homeostasis. Altered glucose metabolism with committed glycolysis addiction is a hallmark of cancer proliferation and viability; therefore, we investigated the effects of designer diets on cancer development in a xenograft model. Three groups of mice were fed with either a low-fat diet, characterised by a high ratio of saturated to unsaturated fatty acids (SFA), or the same diet, in which casein was replaced by free EAAs (SFA-EAA). As a further control for the specificity of the EAA formulation, mice were also fed with a diet, in which casein was substituted with its free amino acids (SFA-CAA) [36] (see par. 2.1).

**Figure 1:**
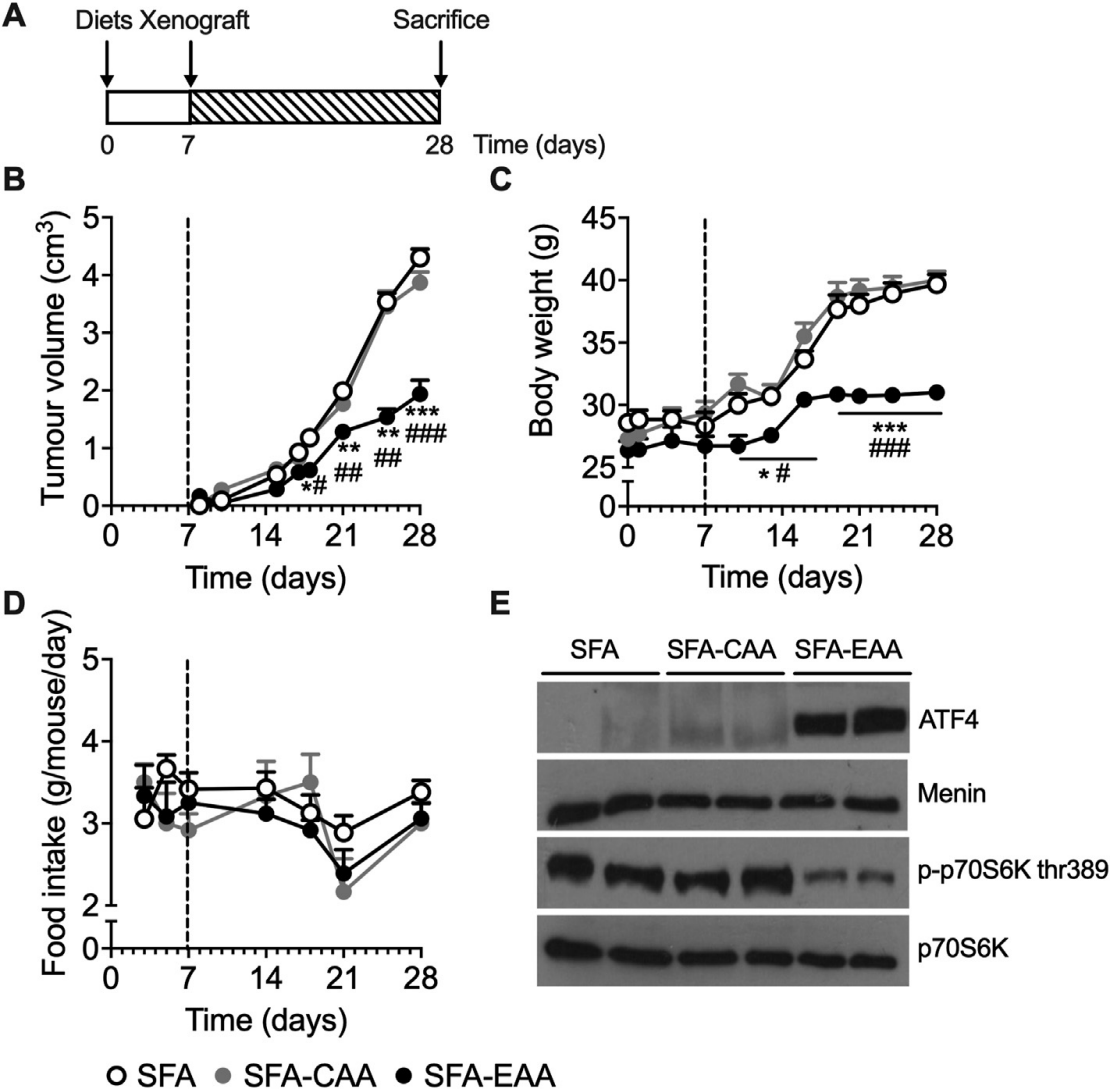
**SFA-EAA diet blocks tumour progression and alter intratumor AA-sensing pathways.** (A) Experimental design. Mice were fed with SFA (n = 10), SFA-CAA (n = 10) or SFA-EAA (n = 10) diet for one week and then xenografted (day 7) with B16F10 melanoma cells. (B) Tumour mass growth over three weeks. (C) Body weight and (D) food intake of the same animals during the same time interval. Mean ± SEM. ∗*P* < 0.05, ∗∗*P* < 0.01, and ∗∗∗*P* < 0.001 vs. SFA diet; ^#^*P* < 0.05, ^##^*P* < 0.01, and ^###^*P* < 0.001 vs. SFA-CAA diet. One-way ANOVA followed by Tukey's post hoc test.

After one week from the start of diets, each group was injected with the highly tumorigenic B16F10 melanoma cell line and tumour development was measured for the subsequent three weeks, after which animals were sacrificed for intratumor analysis (Figure 1A). As shown in Figure 1B, starting at day 10 from tumour injection, tumour mass was already significantly lower (*P* < 0.05) in mice fed with the SFA-EAA diet compared to animals fed with the SFA diet (Figure 1B); within day 14, tumour volume was further reduced in the SFA-EAA group compared to SFA group (∼42%). Food intake (Figure 1D) was slightly decreased, with no statistical significance at any time point, while body weight was reduced by ∼14% after three weeks in the SFA-EAA-fed animals (Figure 1C). Of note, tumour volume was already significantly reduced before any difference in body weight between the two groups, thus ruling out weight loss as a possible cause for the differences in tumour mass.

Notably, the SFA-CAA fed animals did not show any changes neither in tumour volume nor in body weight, as compared to the SFA diet group; this latter finding, besides confirming our previous data [36], also confirmed that the effect of the defined diet on tumour growth was due to the specific AA formulation of the SFA-EAA diet.

We then focused on the possible mechanisms underlying tumour growth inhibition in SFA-EAA-fed animals. The SFA-EAA diet has been shown to significantly affect energy metabolism and energy expenditure [36] by targeting main cell metabolic pathways, such as mitochondrial brown adipose tissue thermogenesis and mTORC1. We, therefore, analysed p70S6K phosphorylation status, as a readout of mTORC1 activity, on tumour explants collected at the end of the experiments. As shown in Figure 1E, p70S6K phosphorylation levels were reduced in xenografts of SFA-EAA mice compared to SFA and SFA-CAA diet-fed animals, indicating inhibition of the mTORC1 pathway. Since this data is at odds with the prominent role of amino acids as mTORC1 activators, we analysed the GCN2-ATF4 pathway, the other primary AA cellular sensor [22]. To this end, we assessed the ATF4 expression on nuclear extracts of tumour explants. As shown in Figure 1E, ATF4 expression was strongly induced in the SFA-EAA diet group compared with both the SFA and SFA-CAA diet-fed animals. These data suggest that the effect of the SFA-EAA diet on tumours is an extensive rearrangement of the intratumor amino acid-sensing pathway, which leads to ATF4 activation, mTORC1 inhibition, and impairment of tumour growth.

### 3.2 A designer diet-based EAA mixture induces apoptotic cell death, specifically in cancer cell lines

In order to investigate more deeply the mechanism underlying the tumour growth inhibition by the amino acid mixture in the SFA-EAA diet, we performed *in vitro* experiments by supplementing both cancer and non-cancer cell lines with an essential amino acid mixture (EAAm). The amino acid composition of the mixture reproduces the stoichiometric ratio of the free AAs in SFA-EAA defined diet, which, in turn, is based on the blend of AAs that has been previously shown when supplemented in drinking water to extend the average life span in mice [30]. The final calculated concentrations of amino acids in the supplemented culture media, at the percentage used (1% w/v), vary from 0.1 to 10 mM, which are in the range of those previously found in mouse Ehrlich ascites cells, and reproduce the high AA content of the tumour environment [41]. In addition to culture media, an amino acid mixture equivalent to that of casein amino acids, in the same range of concentration of EAAm (1% w/v), was also employed as a further control (CAAm).

**Figure 2:**
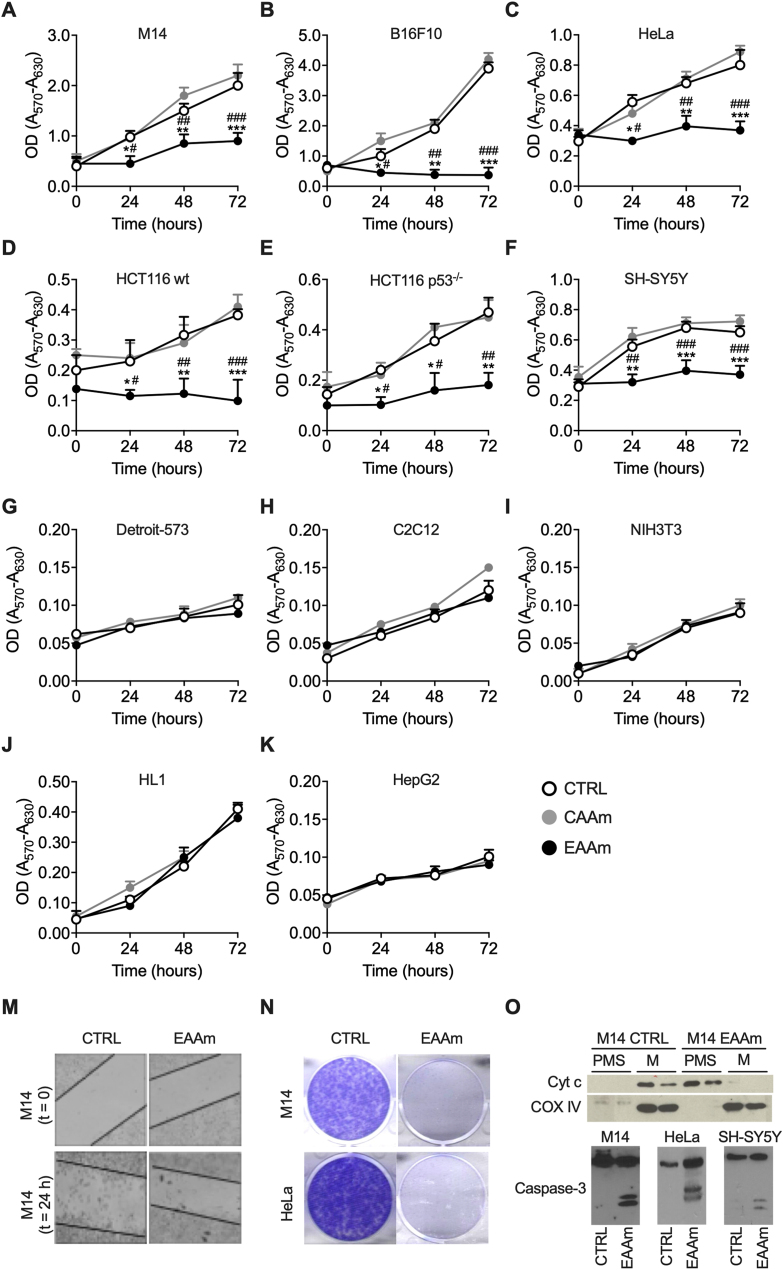
**EAAm mixture inhibits proliferation and induces apoptosis specifically in cancer cells** (A–K) MTT assay on various cancer (A–F) and non-cancer (G–K) cell lines. Cells were seeded in 96 wells and, after 16 h, incubated in culture medium only (CTRL), culture medium supplemented with 1% EAAm mixture (EAAm), or with a casein-based AA mixture (CAAm) (1%). Afterwards, viability was assessed in both control and treated cells by performing the assay at the indicated times. Mean (n = 4) ± SEM ∗P < 0.05, ∗∗P < 0.01, and ∗∗∗P < 0.001 vs. CTRL; #P < 0.05, ##P < 0.01, and ###P < 0.001 vs. CAAm. One-way ANOVA followed by Tukey's post hoc test. (M) Wound healing assay in B16F10 melanoma cells. Cells were seeded and allowed to attach for 16 h. After that, cells were scratched and incubated with DMEM (CTRL) or EAAm supplemented medium (EAAm) for 24 h. (N) Colony formation assay. M14 and HeLa cells were incubated in DMEM (CTRL) or EAAm medium, allowed to form colonies for ten days, and stained. (O) Apoptosis analysis. *Upper panel*: Immunoblot analysis of Cytochrome C (Cyt C) cytosolic translocation. Duplicate samples of mitochondrial (Mito) and Post-mitochondrial-supernatant (PMS) of M14 cells incubated with DMEM or EAAm for 24 h. Samples were probed with anti-CytC or, to show specific CytC translocation, with anti-Cox4 antibody. *Lower panel*: Western blot analysis of Caspase-3 cleavage in M14, HeLa and SH-SY5Y cells incubated with DMEM (CTRL) or EAAm-supplemented medium for 24 h. Data are representative of at least three replicates of n = 3 samples for each condition.

While EAAm did not affect the growth of healthy (non-cancer) cells (Figure 2G–J), it significantly inhibited the growth of the cancer cells as early as 24 h after starting the treatment, as compared to cells grown in DMEM or CAAm medium (Figure 2A–F). In line with the *in vivo* data, we thus confirmed the specificity of the effect of EAAm also *in vitro*. The mixture was effective in all human cancer cells tested, such as M14 and HeLa (Figure 2A and C), in addition to both wild-type (Figure 2D) and p53^−/−^(Figure 2E) HCT116 cells, with 54%, 47%, 50%, and 58% inhibition at 24 h vs cells grown in culture medium alone, respectively. Importantly, these latter data imply that the mechanism of action of EAAm is independent of cancer p53 status. Human neuroblastoma (SH-SY5Y) cells growth was also inhibited by 43% by EAAm (Figure 2F)

The mixture was also active on mouse cancer cells; at 72 h, EAAm inhibited B16F10 mouse melanoma by 87% (Figure 2B). Of note, the hepatocellular carcinoma-derived cell line HepG2, which is non-tumorigenic *in vivo* [42], was not affected by EAAm treatment (Figure 2K). The same results were also obtained when a Sulforhodamine B, a non-dehydrogenase-based method, was used to measure cell proliferation (Supplementary Fig. 1). Since DMEM and CAAm were equally ineffective on cancer cells viability, DMEM was used as the proper control.

The EAAm treatment also reduced cancer cell migration capacity, as assessed by scratch-wound assay in B16F10 melanoma cells (Figure 2M). Furthermore, the EAAm mixture also completely blocked clone formation ability in both M14 and HeLa cells when the single-cell clonogenic potential was challenged in a colony formation assay (Figure 2N).

To assess if the reduction in cell proliferation observed was due to a block in cell division or to induction of cell death, we measured various apoptotic markers. Immunoblot analysis showed evident cytochrome C translocation after 24 h of EAAm treatment in M14 melanoma cells (Figure 2O). Moreover, 24 h of EAAm treatment also induced cleavage of caspase 3 in M14, HeLa, and SH-SY5Y cells (Figure 2O), thus confirming the apoptotic cell death by EAAm supplementation.

### 3.3 EAAm downregulates the mTORC1 pathway and induces ATF4 and ER stress in cancer cells

**Figure 3:**
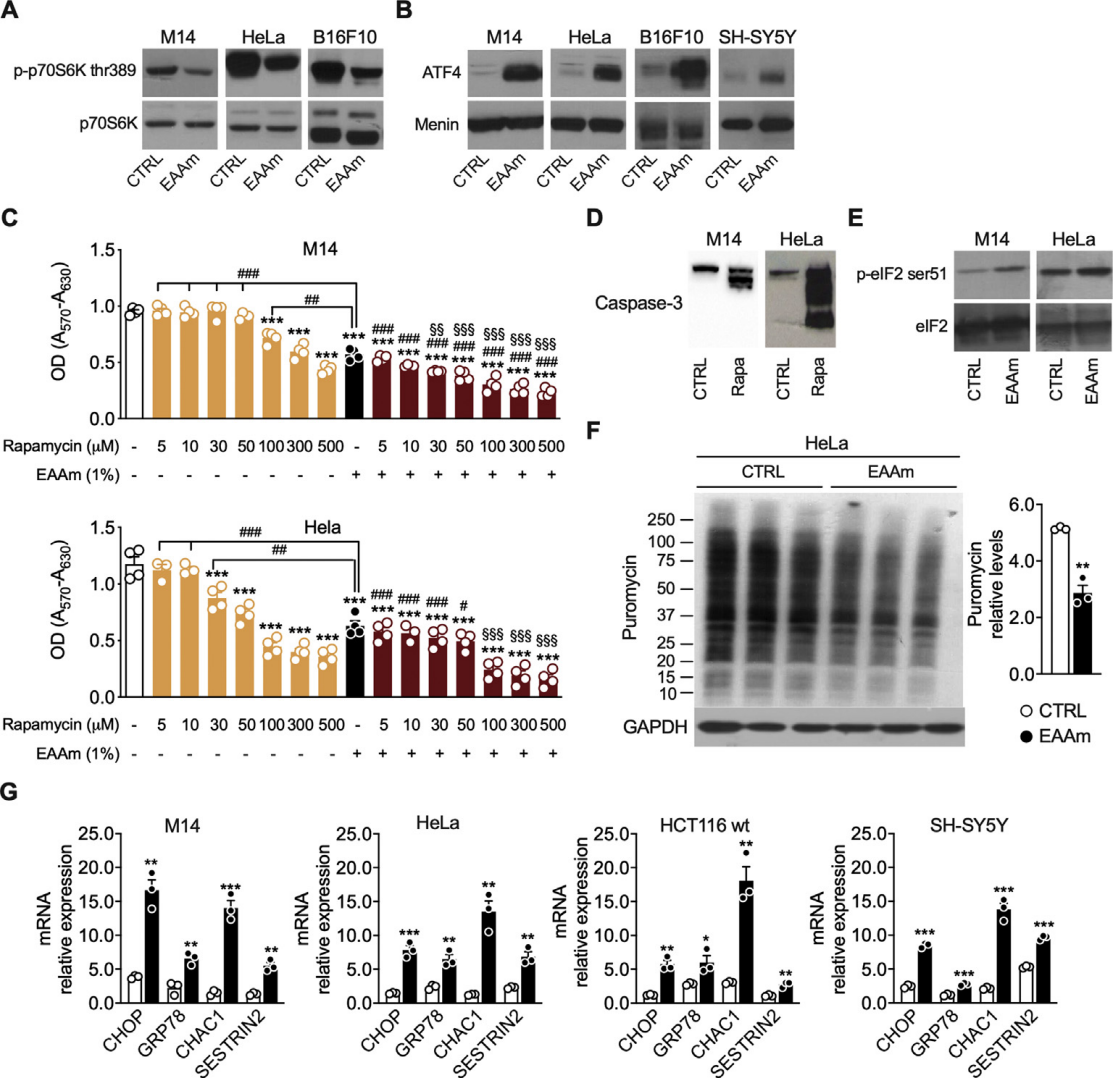
**mTOR pathway inhibition and ER stress induction in EAAm-supplemented cancer cells.** Western blot of phospho p70S6K (p-p70S6K) (A), ATF4 (B), cleaved caspase-3 (D) and p-eIF2 (E) levels in nuclear extracts (ATF4) and whole-cell lysates (p70S6K, p-eIF2 and caspase-3) of the indicated cancer cell lines incubated in culture medium alone (CTRL) or 200 µM rapamycin (Rapa) for 24 h (D), or supplemented with EAAm mixture for 3 h (EAAm). Menin was used as a nuclear loading control. (C) MTT assay of the viability of M14 (above) and Hela (below) cells untreated or treated with rapamycin alone or plus 1% EAAm with the indicated doses for 24 h. Mean (n = 4) ± SEM. ∗∗∗*P* < 0.001 vs. untreated, ^###^*P* < 0.001 vs. rapamycin, ^§§^*P* < 0.01 and ^§§§^*P* < 0.001 vs. EAAm. One-way ANOVA followed by Tukey's post hoc test. (F) Left: SUnSET puromycin labelling immunoblot assay in triplicate samples of HeLa cells incubated with DMEM (CTRL) or the EAA mixture (EAAm) for 5 h. GAPDH is used as a loading control Right: quantification of blot signal levels. ∗∗*P* < 0.01 vs. CTRL Unpaired Student's t-test (G) mRNA expression of ER stress markers in various cancer cells left untreated (CTRL) or incubated for 5 h with the 1% EAAm mixture (EAAm). Mean ± SEM (n = 3) from at least three experiments. ∗*P* < 0.05, ∗∗*P* < 0.01 and ∗∗∗*P* < 0.001 vs. CTRL. Unpaired Student's t-test.

To confirm that apoptosis of cancer cells was also associated with inhibition of mTORC1 signalling and ATF4 upregulation, we analysed phosphorylation of p70Ss kinase and the expression of ATF4 protein in M14 and Hela cells. As shown in Figure 3, 3 h of EAAm treatment were sufficient to downregulate the phosphorylation of p70S6K (Figure 3A) and induce ATF4 protein expression (Figure 3B) in several different cancer cell lines. In line with the lack of effects on viability, phosphorylation of p70S6K and ATF4 levels were not affected by EAAm in both non-cancerous cells (Detroit 573) and the EAAm-resistant HepG2 cell line (Supplementary Figs. 2A and B). These results strongly suggest that EAAm inhibits mTORC1, activates ATF4, and causes apoptosis specifically in cancer cells.

Then, we treated both M14 and Hela cells with the mTORC1 inhibitor rapamycin and assessed their viability to validate this point further. As shown in Figure 3C, rapamycin up to 10 or 50 μM did not affect cell viability in Hela and M14 cells; however, higher concentrations dose-dependently reduced proliferation of both cell lines. This result is in line with early reports which show that the effect of rapamycin on cancer cell viability is strongly dependent on cell type, and high doses are required for apoptosis induction [43,44]; accordingly, 200 μM rapamycin displayed a clear caspase-3 cleavage in M14 and Hela cells (Figure 3D). However, EAAm supplementation to rapamycin-treated cells significantly potentiated its effects, leading to inhibition in cell proliferation even at the lowest doses (Figure 3C). These data, therefore, confirm that EAAm simultaneously targets ATF4 and mTORC1, leading to sensitization of cancer cells to the toxic effects of rapamycin, thus increasing the drug's efficacy.

ATF4 protein levels are regulated by the eIF2 translation initiation factor, whose activity, as a part of the integrated stress response (ISR), is induced by ER stress ([23,24]). To verify if the increase in ATF4 protein expression in EAAm-treated cancer cells resulted from the induction of the ER stress, we first performed immunoblot analysis of eIF2 phosphorylation status in cells treated with the EAAm mixture. As shown in Figure 3E, 3 h of EAAm treatment increased phospho-eIF2 levels in HeLa and M14. Furthermore, in line with the increased eIF2 phosphorylation and inhibition of mTORC1 signalling in EAAm treated cells, global protein synthesis was reduced after 5 h of EAAm treatment in HeLa cells (Figure 3F).

In keeping with these results, the mRNA levels of several ER stress markers, such as CHOP, GRP75, CHAC1, and Sestrin2, were also upregulated by the EAAm treatment in cancer cell lines (Figure 3G). On the contrary, the ER stress markers were not affected (or partly downregulated) by the EAAm treatment in non-cancer Detroit 573 fibroblasts (Supplementary Fig. 2C). Importantly, the EAAm supplementation did not induce ER stress markers expression in EAAm-resistant HepG2 cells, thus underscoring the association between ER stress induction and cell death (Supplementary Fig. 2C). Overall, these data show that the EAAm mixture inhibits mTORC1 and activates ATF4 and ER stress exclusively in cancer cells.

### 3.4 Metabolomic analysis shows a decline in some NEAA, as well as in cell bioenergetic status, in EAAm-treated cells

**Figure 4:**
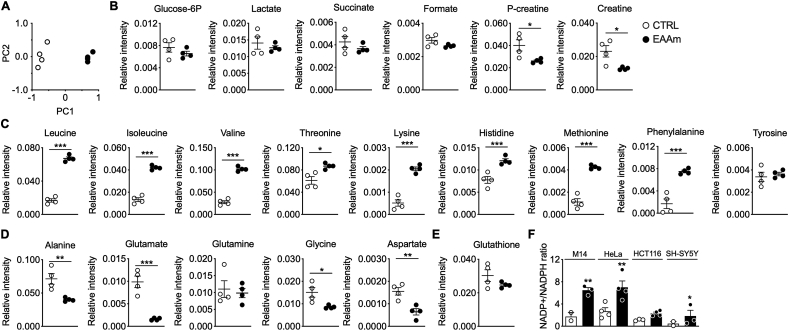
**NMR metabolite analysis in EAAm-treated M14 melanoma cells shows changes in energy-metabolism intermediates and NEAA levels.** Cells were untreated (CTRL) or incubated with EAAm for 5 h (EAAm). (A) Principal component analysis (PCA) between CTRL (n = 4) and EAAm (n = 4) cells. (B–E) Amino acid and metabolite levels in control and treated cells. Mean ± SEM. ∗P < 0.05, ∗∗P < 0.01 and ∗∗∗P < 0.001 vs. CTRL. Non-parametric Wilcoxon test. (F) NADP^+^/NADPH ratio in cells incubated with culture medium (CTRL) or supplemented with 1% EAAm (EAAm) for 5 h (n = 4) Mean ± SEM. ∗*P* < 0.05 and ∗∗*P* < 0.01 vs. CTRL. Unpaired Student's t-test.

To investigate how EAAm supplementation activated the eIF2-ATF4 axis of ER stress, we performed [^1^H]-NMR untargeted metabolomic analysis in M14 cells treated with EAAm mixture for 3 h. Principal component analysis (PCA) revealed net clusterization between control and EAAm treated cancer cells (Figure 4A), thus indicating that the EAAm supplementation leads to remarkable changes in metabolite levels and a significant reprogramming of cell metabolic pathways, also in line with the data on altered amino acid sensing pathways (Figure 1E, Figure 3A, Figure 3B). Remarkably, the most affected metabolic substrates were those related to glycolysis − glucose-6-phosphate (G6P) and lactate levels showed a trend toward a decrease in the EAAm-treated cells (Figure 4B), thus suggesting downregulation of glycolytic flux. Formate levels were also diminished, albeit not significantly, in the EAAm-treated cells, indicating perturbation of the one-carbon metabolism pathway [45], while downregulation of both creatine and phospho-creatine is indicative of a bioenergetic failure [46] (Figure 4B).

Intracellular levels of all the AA present in the EAAm mixture were increased, confirming an active cellular uptake of all exogenous amino acids (Figure 4C). In net contrast, the endogenous levels of the four non-essential amino acids (NEAA), glutamate (Glu), glycine (Gly), aspartate (Asp), and alanine (Ala), were decreased after EAAm treatment (Figure 4D). In contrast, the remaining amino acids showed no change in their intracellular abundance (data not shown). Accordingly, total glutathione, which is synthesized, among others, from Gly and Glu as precursors, also showed a trend toward decreased levels in EAAm treated cells, thus suggesting diminished biosynthesis, as well as a drop in antioxidant status. (Figure 4E).

To further investigate this point, we measured the ratio of oxidized (NADP^+^) vs reduced (NADPH) nicotinamide-adenine-dinucleotide phosphate in different cancer cell lines after 5 h of EAAm supplementation. In line with the data on glutathione, the ratio of oxidized vs reduced nicotinamide-adenine-dinucleotide phosphate (NADP^+^/NADPH) levels was increased in the EAAm-supplemented cells (Figure 4F). This result further indicates a fall in intracellular redox potential, in line with the reduction in G6P (Figure 4B), which presumably also reflects a decrease in the pentose-phosphate pathway branch of glycolysis, a major NADPH producing pathway.

The EAAm supplementation, similarly to consumption of the SFA-EAA diet, affected the major AA-sensing effectors; as such, the drop in NEAA levels induced by EAAm was particularly intriguing. Therefore, we extended our analysis by measuring total AA levels in plasma and tumour explants of SFA-, SFA-EAA-, and SFA-CAA-fed mice. The measurement of AA levels in tumour explants confirmed the decrease in Glu, but not Asp, Gly, or Ala levels, in SFA-EAA-fed mice, compared to both SFA- and CAA-fed animals (Supplementary Fig. 3). Additionally, the intratumor amounts of the three BCAA (*i.e.*, Leu, Ile and Val) and those of threonine (Thr) were also increased by SFA-EAA feeding, thus reproducing their intracellular aminogram profiles. On the contrary, tyrosine (Tyr) levels decreased in tumours but not in cells, while cystine (Cys) increased in tumour explants obtained from mice fed with the SFA-EAA diet, and its levels were undetectable in cells (compare Figure 4 with Supplementary Fig. 3). The circulating AA levels showed an increase in Leu, Val, Thr and Tyr in SFA-EAA-fed mice compared to both SFA- and SFA-CAA-fed mice, thus indicating a different profile as compared to both intracellular and intratumor aminograms (Supplementary Table 2). However, supplementation of plasma extracts to M14 cells did not affect their viability (Supplementary Fig. 5).

Altogether, these data indicate that treatment of cancer cells with the EAAm leads to profound changes in cancer cells’ metabolites, suggesting an alteration of substrate utilization. This metabolic alteration is reflected by intracellular depletion of several metabolites linked to glucose metabolism, redox homeostasis, as well as of specific NEAAs.

### 3.5 The reduction in Glu levels by EAAm leads to ATF4-dependent mTORC1 inhibition and underlies the EAAm-dependent induction of cell death

**Figure 5:**
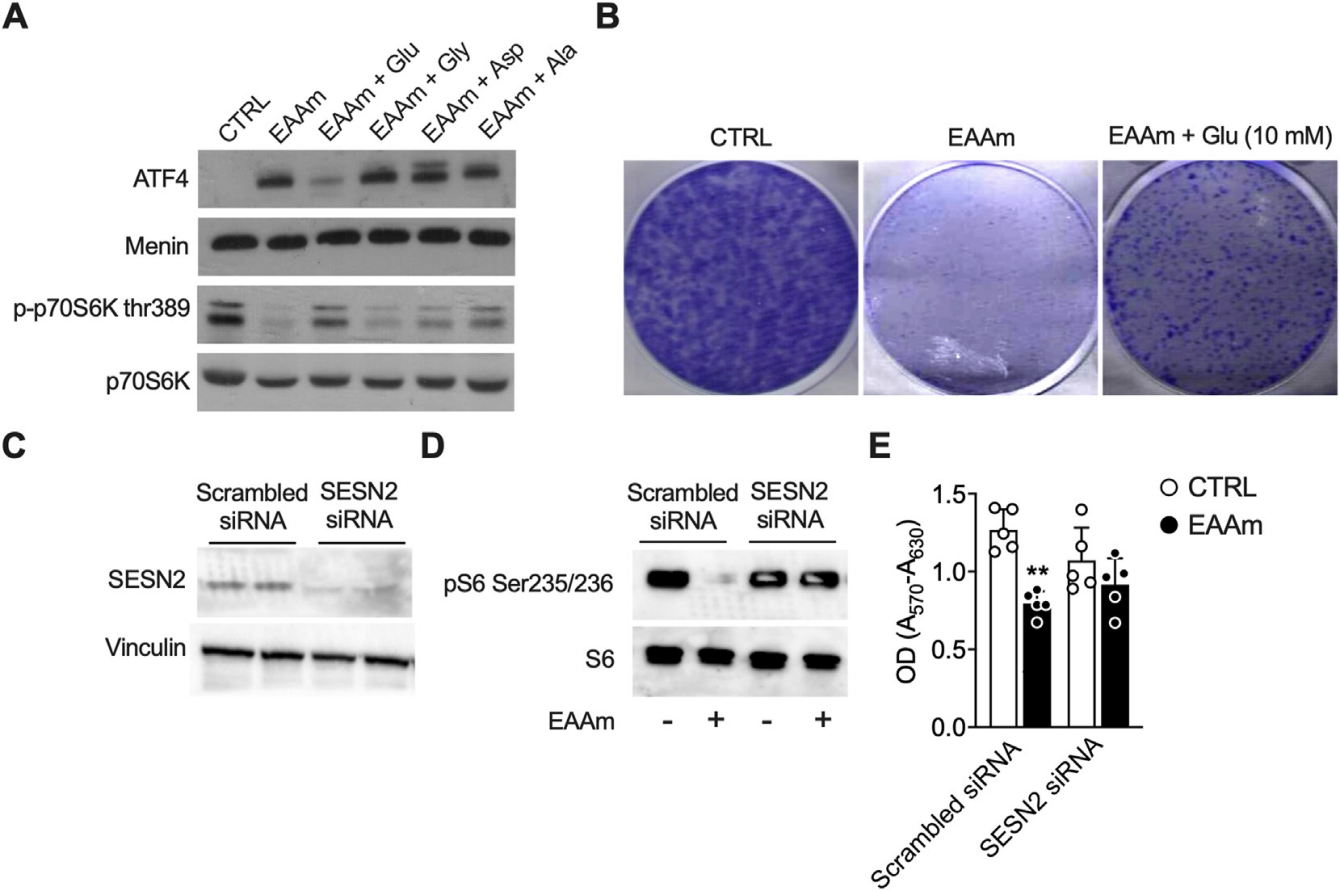
**Glu supplementation blunts the effects of EAAm on ATF4 induction, mTOR inhibition and cell growth**. (A) Western blot analysis of nuclear extracts (ATF4) or total lysates (p70S6K) of M14 untreated (CTRL), incubated with EAAm mixture, or incubated with EAAm mixture plus Glu, Gly, Asp or Ala 10 mM for 3 h. (B) Clonogenic assay in M14 cells left untreated (CTRL), incubated with EAAm mixture (EAAm), or incubated with EAAm mixture plus Glu 10 mM (EAAm + GLU) for 24 h (C) sestrin2 (SESN2) protein expression in HeLa cells transfected with non-targeting (scrambled) or SESN2- specific siRNA. Vinculin is shown as a loading control (D). p-S6 levels immunoblot analysis in Hela cells transfected with control non-targeting siRNA (scrambled), or siRNA against sestrin2 (SESN2), and treated (+) or not (−) with EAAm 1%.for 3 h (E) MTT assay of Hela cells transfected with non-targeting (scrambled) siRNA or siRNA against sestrin 2 (SESN2) and incubated with culture medium (CTRL) or supplemented with 1% EAAm for 24 h (EAAm). Mean (n = 4) ± SEM, ∗∗*P* < 0.01 vs. CTRL. Two-way ANOVA followed by Tukey's post hoc test.

ATF4 and mTORC1 are activated and inhibited, respectively, by amino acid starvation [47]. The drop in Glu, Gly, Asp, and Ala levels induced by EAAm supplementation could therefore explain their regulation by the mixture. If so, then adding back the four AAs should rescue both ATF4 and mTORC1 regulation, likewise the EAAm-induced cell death. To verify this hypothesis, we measured ATF4, p-p70S6K levels, and clonogenic ability in cancer cells treated with EAAm mixture alone or EAAm mixture supplemented with Glu, Gly, Asp, or Ala. Glu re-addition, but not Gly, Asp or Ala, blunted the ATF4 induction and restored p-p70S6K downregulation induced by EAAm (Figure 5A). Furthermore, adding Glu to the EAAm mixture also partially recovered the clonogenic ability of M14 (Figure 5B). Neither Asp nor Gly or Ala rescued the clonogenic potential, while the addition of Glu, Asp, Gly and Ala together did not further increase the clonogenic potential compared to Glu alone (not shown). Therefore, these results indicate that the shortage of Glu resulting from EAAm treatment activates ATF4, downregulates mTORC1, and induces cell death.

The EAAm supplementation led to increased expression of Sestrin2 (Figure 3G) − which is under the control of ATF4 [48,49] and, by interacting with GATOR complex proteins, acts as an mTORC1 inhibitor [50,51]. To confirm that the EAAm dependent mTORC1 downregulation proceeded through the ER stress ATF4-Sestrin2 pathway, we analysed phosphorylation levels of the mTORC1 downstream target S6 ribosomal protein (S6) in HeLa cells transfected with scrambled or Sestrin2-specific small interfering RNAs (siRNA) and incubated with standard DMEM or EAAm mixture for 3 h. Immunoblot analysis shows that siRNA efficiently reduced Sestrin2 protein expression (Figure 5C). While the EAAm treatment reduced, as expected, the p-S6 levels in cells transfected with the scrambled siRNA, the phosphorylation of S6 was unchanged in the Sestrin2-silenced cells (Figure 5D, *right panel*). This result confirms that inhibition of mTORC1 by EAAm is Sestrin2-dependent. Furthermore, the EAAm-induced loss of viability was partially rescued by Sestrin2 silencing (Figure 5E). Therefore, these findings suggest that the shortage of Glu induced by EAAm supplementation leads to ATF4/Sestrin2 activation, mTORC1 inhibition, and cancer cell death.

### 3.6 EAAm inhibits glycolysis in cancer cells

**Figure 6:**
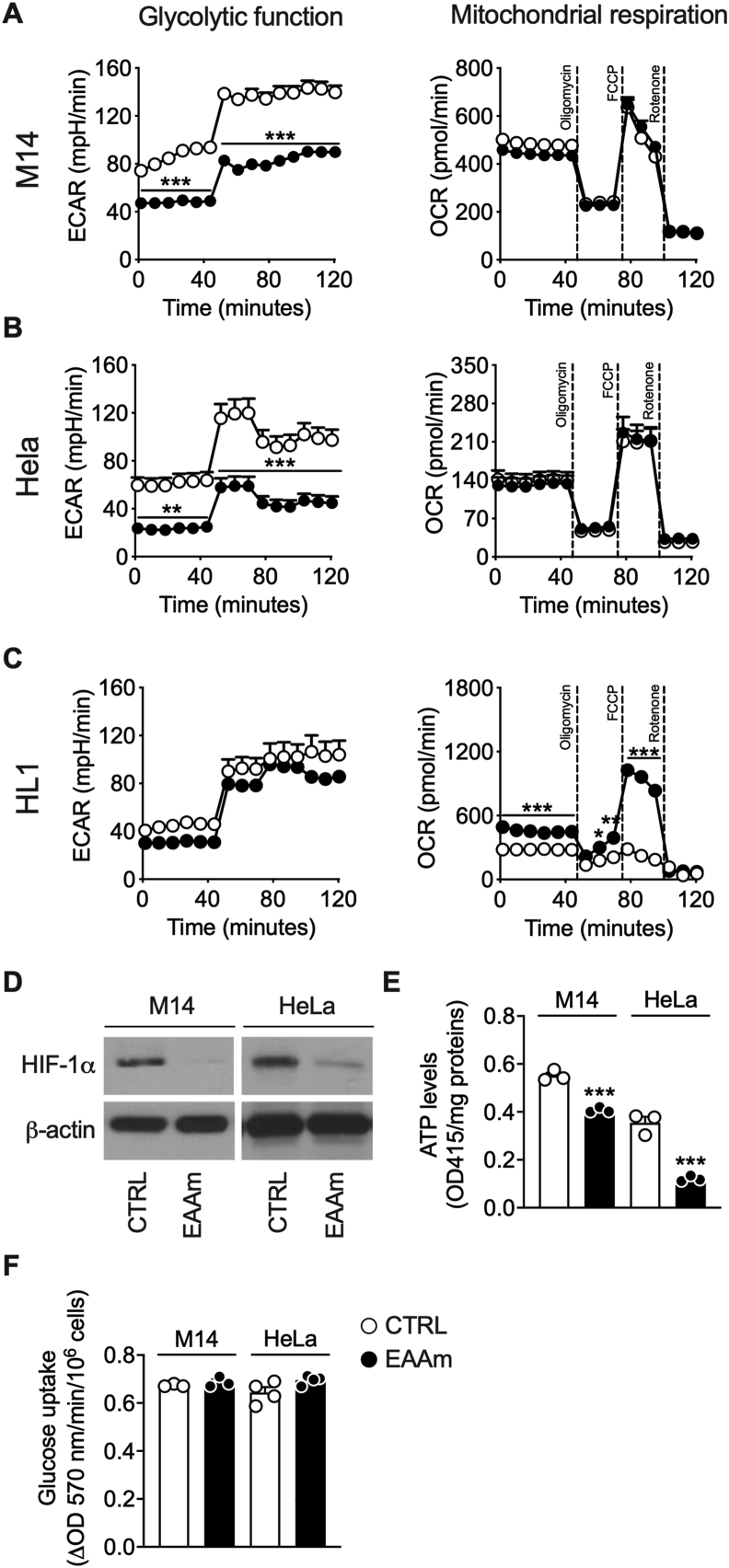
**EAAm supplementation inhibits glycolysis in cancer cells.** Seahorse analysis of ECAR (*left panel*) and OCR (*right panel*) in M14 (A), Hela (B) and HL-1 normal cardiomyocytes (C) incubated in DMEM (CTRL) or EAAm-supplemented (EAAm) media for 1 h. After basal measurement, oligomycin (2 μM), FCCP (1 μM) and rotenone/antimycin A (both 0.5 μM) were injected at the times indicated. Mean (n = 4) ± SEM from at least three experiments. ∗*P* < 0.05, ∗∗*P* < 0.005 and ∗∗∗*P* < 0.001 vs CTRL. Unpaired Student's t-test. (D) Western blot analysis of HIF1α expression in M14 and Hela cells untreated (CTRL) or treated with EAAm for 3 h (EAAm). β-actin is shown as a loading control. (E, F) ATP levels (E) and glucose uptake (F) in both M14 and Hela cells non supplemented (CTRL) or supplemented with 1% EAAm for 5 h. Data are mean (n = 3) (Figure 6E), (n = 4) (Figure 6F) ± SEM ∗∗∗*P* < 0.001 vs CTRL. Unpaired Student's t-test.

Metabolomic data indicate an apparent involvement of NEAAs and glucose metabolic pathways in the EAAm mechanism of action. In particular, alteration of glucose metabolites suggests downregulation of glycolysis. On the other hand, the metabolism of NEAAs is mainly localised in the mitochondrial matrix, where they play a major role in both anaplerotic and cataplerotic reactions of the TCA cycle. Since mice fed with an amino acid-defined diet display a switch toward aerobic substrate oxidation and mitochondrial biogenesis [36], we verified if the inhibition of glycolysis and increase of mitochondrial substrates utilisation could underlie the EAAm-induced cancer cell death. To this end, we measured both extracellular acidification rate (ECAR) and oxygen consumption rate (OCR) in M14 and HeLa cells, and HL-1 cardiomyocytes as a non-cancer cell line control. As shown in Figure 6, in line with the metabolomic data, the EAAm treatment (1 h) decreased basal glycolytic rate in both M14 (Figure 6A) and HeLa cells (Figure 6B).

Furthermore, according to ECAR data, the EAAm treatment also downregulated the expression of hypoxia inducible factor 1α (HIF-1α) in M14 and HeLa (Figure 6D), which, by upregulating glycolysis, is one of the primary regulators of the Warburg effect and cancer growth [52]. ATP levels were also reduced by EAAm supplementation in both M14 and Hela cells, thus confirming a metabolic crisis (Figure 6E). Glucose uptake, however, was unchanged between control and EAAm-treated cells (Figure 6F); this would suggest that the EAAm-induced decrease in ECAR was due to an effect downstream of the glucose uptake step. In line with this hypothesis, the metabolomic data that showed a decrease in glucose-6-phosphate beyond lactate and Ala levels in EAAm-treated cells suggest a decreased glycolytic utilisation of glucose and a reduced production of pyruvate (Pyr). However, supplementation of both EAAm-treated Hela and M14 cells with exogenous Pyr failed to rescue the EAAm-induced cell death in a clonogenic assay (Supplementary Fig. 4), even if it should be noted that an excess of Pyr has been found to inhibit lactate dehydrogenase [53]. Accordingly, Pyr reduced the clonogenic ability also in cells supplemented with the control medium (Supplementary Fig. 4). Notably, in both M14 and HeLa cells, OCR was not increased by EAAm treatment, thus indicating that these cells cannot adapt appropriately when provided with an alternative oxidative metabolic substrate. In fact, in contrast with M14 and HeLa, the EAAm treatment displayed a slight, non-significant decrease of ECAR beyond a marked increase of OCR in the non-cancer HL-1 cells (Figure 6C), in line with our previous data [30].

**Figure 7:**
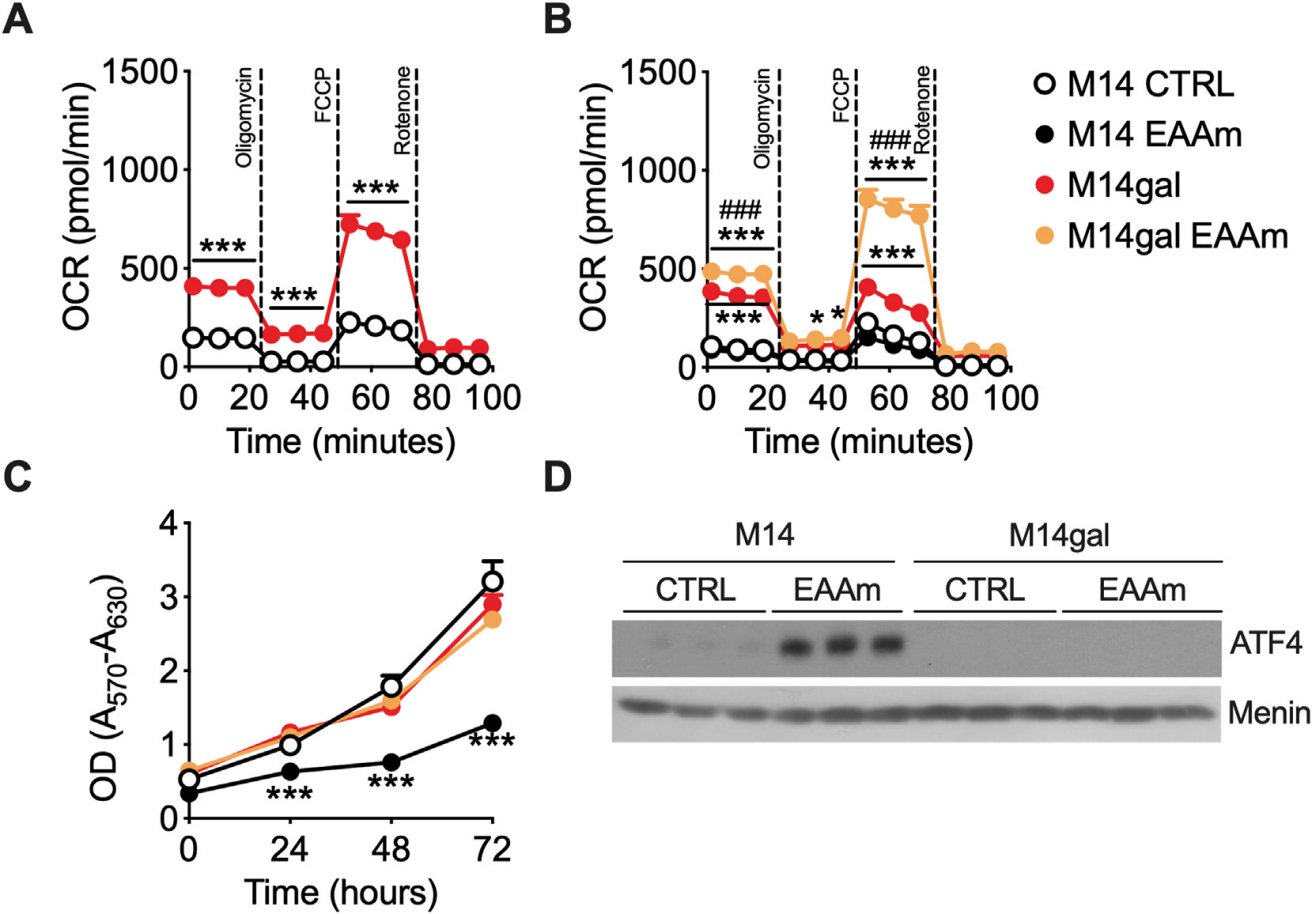
**Oxidative phenotype in M14 melanoma cells provides resistance to EAAm.** (A) OCR in M14 and galactose-adapted M14 cells (M14gal) (B) OCR in M14 and M14gal cells untreated (CTRL) or incubated in EAAm mixture for 1 h (EAAm). After basal OCR measurement, drugs were injected as in Figure 6. Panel A: mean (n = 3) ± SEM, ∗∗∗*P* < 0.001 vs CTRL (Unpaired Student's t-test). Panel B: mean (n = 3) ± SEM ∗∗∗*P* < 0.001 vs CTRL ^###^*P* < 0.001 vs M14gal (two-way ANOVA followed by Tukey's post hoc test). (C) MTT viability assay of M14 and M14gal cells treated (EAAm) or not (CTRL) with EAAm mixture and analyzed at the indicated time points Mean (n = 4) ± SEM ∗∗∗*P* < 0.001 vs CTRL (two-way ANOVA followed by Tukey's post hoc test). (D) Western blot analysis of ATF4 expression in triplicate samples of nuclear extracts of M14 and M14gal untreated (CTRL) or treated with EAAm 1% for 3 h (EAAm). Menin is shown as a nuclear loading control.

To confirm that glycolysis addiction and lack of metabolic flexibility in response to an oxidative stimulus underlies the sensitivity of cancer cells to the EAAm mixture, we measured bioenergetic profile and viability in M14 cells in which proliferation rate was uncoupled from glycolytic dependency. To this end, cells were subjected to several rounds of cultivation and adaptation in galactose medium, which switches their metabolic activity toward oxidative phosphorylation (OXPHOS) at the expense of aerobic glycolysis [54]. Galactose-adapted M14 cells (M14gal) displayed increased basal and maximal OCR compared to M14 (Figure 7A), confirming the oxidative metabolic switch of these cells with respect to parental M14 cells. Furthermore, as shown in Figure 7B, in contrast to M14, M14gal cells significantly increased OCR in response to EAAm treatment, while ECAR was not affected. Although the proliferation of M14 was, as expected, significantly reduced by EAAm in all-time points (Figure 7C, see also Figure 2A), the viability of oxidative M14gal cells was not affected by the EAAm treatment. In line with this, ATF4 protein induction in M14 by EAAm was absent in the non-glycolytic M14gal (Figure 7D), thus indicating that glycolysis inhibition by the mixture was responsible for ATF4 induction/mTORC1 inhibition and cell death of cancer cells.

Overall, these data suggest that, due to these cells’ lack of metabolic flexibility in response to an alternative energy substrate, the metabolic reprogramming induced by our EAAm formulation is specifically toxic to cancer cells, presumably because of their inability to adapt to oxidative/mitochondrial metabolic substrate supply.

### 3.7 EAAm mixture activates BCAA oxidation

**Figure 8:**
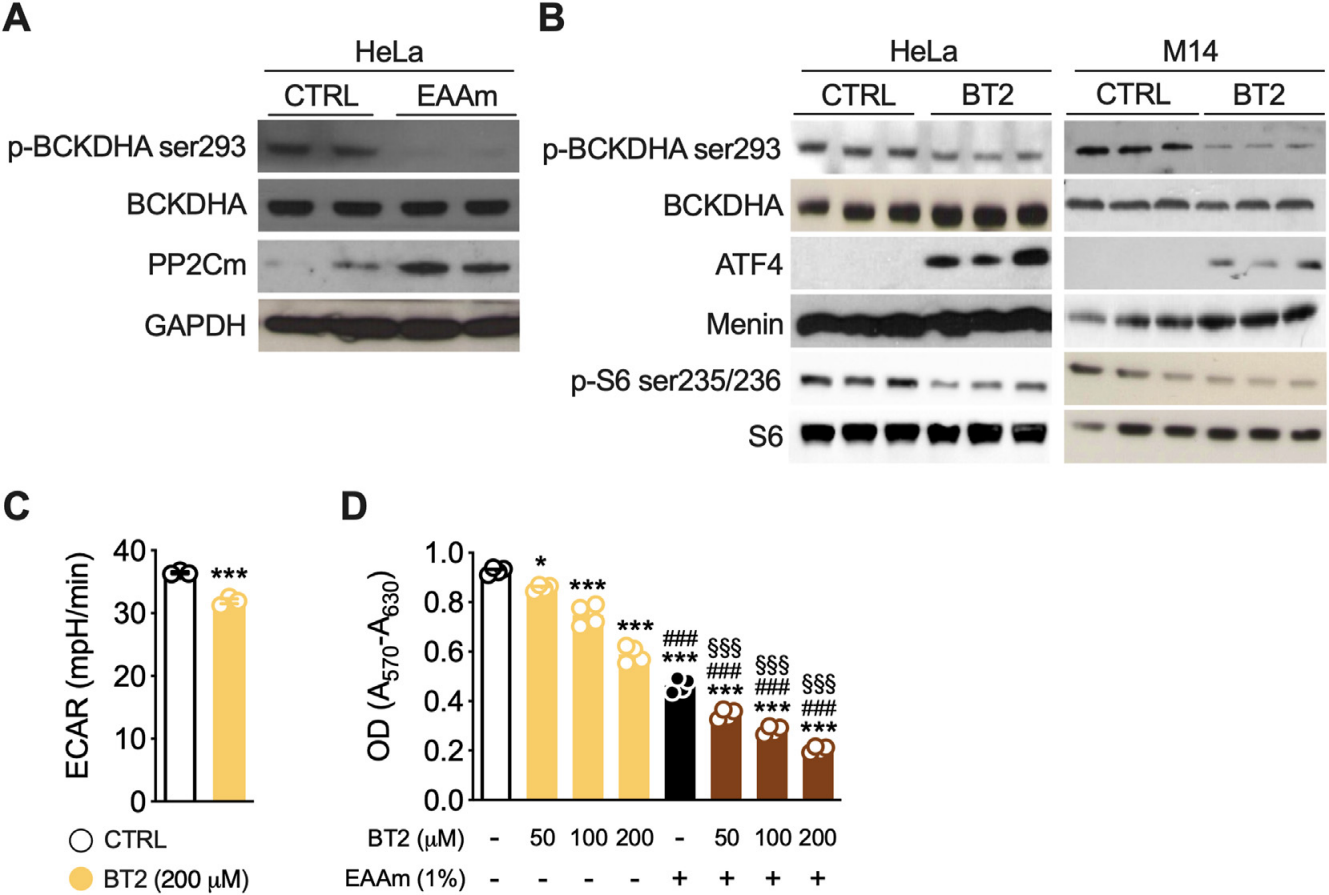
**EAAm activates BCAA catabolism, and BT2 potentiates the effects of EAAm** (A) Expression of PP2Cm and phosphorylation levels of BCKDHA dehydrogenase in Hela cells left untreated (CTRL) or treated with or EAAm (EAAm) for 3 h (B) phospho-BCKDHA dehydrogenase, phospho-S6 ribosomal protein and nuclear expression of ATF4 in HeLa (left) and M14 (right) cells treated with vehicle (CTRL) or with the BCKDK inhibitor BT2 (100 μM) for 5 h. Menin is shown as a nuclear loading control for ATF4. (C) Glycolysis rate (ECAR) in M14 cells vehicle-treated (CTRL) or treated with BT2 200 μM for 1 h (D) MTT assay in M14 cells treated with vehicle (CTRL) or treated with BT2 or BT2 plus EAAm for 16 h with the indicated doses. Mean (n = 4) ± SEM from at least two experiments. ∗*P* < 0.05 and ∗∗∗*P* < 0.001 vs. CTRL, ^###^*P* < 0.001 vs. BT2, ^§§§^*P* < 0.001 vs. EAAm. Two-way ANOVA followed by Tukey's post hoc test.

Our EAAm mixture inhibited glycolysis in cancer cells and increased mitochondrial OCR in non-cancer and oxidative cancer cells. Therefore, we investigated which mitochondrial pathway was affected by the EAAm. Since EAAm is enriched in BCAA, we verified if EAAm could activate the mitochondrial BCAA oxidation pathway by assessing the regulation of its catabolic enzymes. BCAA are catabolized by the concerted activity of branched-chain aminotransferase (BCAT) and branched-chain α-ketoacid dehydrogenase complex (BCKDHA) enzymes [55]. This latter's activity is regulated by its inhibitory kinase BCKDK and activating mitochondrial protein phosphatase 2C (PP2Cm) [56,57]. As shown in Figure 8A, 1 h of EAAm mixture treatment increased the expression of PP2Cm protein in HeLa cells, which was associated with decreased levels of p-BCKDHA, thus confirming the activation of the BCAA catabolic pathway by the mixture.

To further verify that the effects of EAAm on cancer cells viability, glycolysis inhibition, ER stress induction, and mTORC1 inhibition were due to the activation of BCAA catabolism, we treated M14 and Hela cells with the BCKDK inhibitor BT2 [58]. As expected, and similarly to EAAm, BT2 treatment activated the BCAA catabolic pathway, as indicated by decreased levels of phospho-BCKDHA in both M14 and HeLa cells (Figure 8B). Most importantly, BT2 treatment downregulated ECAR (Figure 8C) and induced ATF4 expression, in addition to downregulating the mTORC1 pathway in both M14 and HeLa cells, as indicated by reduced phosphorylation of S6 ribosomal protein (Figure 8B). Furthermore, BT2 not only dose-dependently inhibited M14 viability, but its effects were significantly potentiated by EAAm, thus indicating a shared mechanism of action (Figure 8D).

To additionally validate these results and exclude off-target effects of BT2, we measured both ECAR and cell proliferation in BCKDK-silenced M14 cells (Supplementary Fig. 6). As expected, BT2 decreased glycolytic rate in scrambled siRNA-transfected cells (Supplementary Fig. 6B). However, the effective BCKDK silencing (as evidenced at the protein level) (Supplementary Fig. 6A) blocked the BT2-downregulated ECAR (Supplementary Fig. 6B), BT2 reduced, and EAAm further inhibited cell viability in the scrambled siRNA-transfected M14 cells, while on the contrary either treatment was ineffective in reducing BCKDK-silenced cell proliferation (Supplementary Fig. 6C). Of note, both ECAR and cell viability were lower already in control, untreated BCKDK-silenced than scrambled M14 cells, thus confirming the harmful effects of BCKDK inhibition on glycolysis and cell growth. Therefore, these results suggest that, at least under our experimental conditions, the mechanism of action of BT2 and EAAm in M14 cells is BCKDK-dependent. Overall, BT2 recapitulated the effects of EAAm on cancer cells, thus suggesting that activation of BCAA catabolism may underlie the mixture's effects on glycolysis, ATF4 expression, mTORC1 signalling, and cancer cell death.

## 4 Discussion

We show here that a diet in which protein component was substituted with a defined formula of EAAs (SFA-EAA diet) blocks cancer growth in mice. Moreover, our findings demonstrated that a similar AA mixture (EAAm) reduces glycolysis and inhibits mTORC1 in cancer cells. Notably, the EAAm selectively promoted an unresolved ER stress and apoptotic death in cancer but not non-cancer cells. Aerobic glucose utilization is a hallmark of cancer cell proliferation, and several glycolysis inhibitors are currently in the clinical development stage [59,60]. Our findings suggest that the simultaneous consumption of such amino acid designer diet and glycolytic inhibitors treatment − combining their effects − might represent a novel, promising therapeutic strategy for cancer.

We have previously shown that the SFA-EAA diet prevented and reduced body weight gain in rodent models of dietary and genetic obesity by increasing energy expenditure through activating brown adipose tissue thermogenesis. Mechanistically, the amelioration of the glycaemic profile of obese mice and the increase in mitochondrial substrate oxidation pointed to an improvement of substrate selection and utilization by the designer diet, increasing metabolic flexibility. Our present work suggests that targeting this same process in cancer cells, with modification of substrate supply, leads to specific tumour growth impairment both *in vivo* and *in vitro*.

The pivotal role of AAs in increasing mitochondrial biogenesis and activity is now well-established [30,61]. In line with this, and extending these findings, activation of mitochondrial BCAA catabolism (Figure 8) confirmed the EAA involvement in mitochondrial stimulation. This mechanism could explain the inhibitory effects of EAAm on the glycolytic pathway: a reciprocal negative regulation between mitochondrial respiration and glycolysis is, in fact, well-known (*i.e*., the Crabtree, Pasteur, as well as Warburg effect itself), with fatty acid oxidation as the primary regulatory pathway. Accordingly, an inverse relationship between amino acid and glucose metabolism has been long described [62,63], and plasma BCAA levels are known to be negatively associated with glucose disposal and are suspected of causing insulin resistance [64]. High glucose levels have been shown to inhibit BCAA oxidation [65], whereas mice deficient in BCAA catabolism show enhanced glucose utilization [66]. Furthermore, metabolomic analysis of highly glycolytic hearts of mice overexpressing a constitutively active form of phosphofructokinase-2 (PFK-2) showed elevated levels of BCAAs [67]. Therefore, BCAA oxidation and glycolysis are reciprocally regulated in the same way as glucose and fatty acid metabolism. However, although conferring a growth advantage, the tumour Warburg effect results in glucose dependency and limited metabolic flexibility. Consequently, activation of mitochondrial BCAA catabolism by the EAAm, by forcing a change in substrate utilization, could selectively impair the viability of the tumour cell by affecting their metabolic addiction and inflexibility. Supporting this hypothesis, the BCKDK inhibitor BT2 recapitulates many of the effects of our AA mixture on cancer cells.

Remarkably, our findings also resemble those of a recent report, which shows that catabolism of BCAA is lost during the progression of cells toward carcinogenesis: this caused an increase in BCAA levels and mTORC1 hyperactivation; however, restoring the BCAA catabolic pathway with BT2 impaired the cancer cell proliferation [68]. Therefore, those and our present data indicate that re-activation of the lost BCAA catabolic pathway in cancer could be a valuable therapeutic strategy. In particular, our findings, which are based on a dietetic approach, are of significant clinical interest. To our knowledge, this is the first report showing inhibition of glycolysis by nutritional activation of the BCAA catabolism in cancer cells.

Although the exact mechanism by which the exposure to EAAm reduced intracellular Glu, Gly, Ala, and Asp levels, leading to ER stress induction in cancer cells, requires further investigation, this effect seems to be a consequence of the EAAm-induced inhibition of glycolysis and Pyr output. Ala is produced from the transamination of cytosolic Pyr through Ala transaminase (ALT) and is, therefore, together with lactate, a by-product of glycolysis. The reduced Ala levels could result from the decrease in the availability of glycolytic Pyr. Accordingly, dichloroacetate (DCA), an inhibitor of pyruvate dehydrogenase kinase, by increasing Pyr delivery for oxidation, has been shown to decrease the production of Ala in both cells [69] and tissues [[70], [71], [72]]. Notably, DCA has long been proposed as an anticancer drug [38].

The inhibition of ALT activity through the EAAm-induced decrease in Pyr availability could then lead to a reduction in α-ketoglutarate (α-KG) that, in turn, would be also less available for Glu synthesis by Glu dehydrogenase (GDH) reaction. In cancer cells, the GDH activity is massively directed toward the reductive biosynthesis of Glu [73], with the purpose of both ammonia recycling and biomass production [74]. Therefore, in EAAm-treated cells, blockade of Ala and α-KG output by the deficiency of glycolytic Pyr would interfere with Glu biosynthesis, ultimately leading to the decrease in Glu amount.

Glycolysis inhibition would also explain the HIF-1α downregulation (Figure 6D), which would be otherwise at odds with an EAAm-induced decrease in α-KG levels, since the HIF-1α protein stability is known to be negatively regulated by α-KG through an oxygen-dependent prolyl-hydroxylases (PHD) [75]. However, under our *in vitro* experimental conditions, the HIF-1α protein levels were abundant even in control, normoxic cells (Figure 6D), thus suggesting a PHD- and oxygen-independent regulation. Lactate and Pyr accumulation promote the HIF-1α stabilization in cancer cells [76,77]. Therefore, the EAAm-dependent reduction of HIF-1α could be due to the EAAm-inhibited glycolysis. The drop in Asp and Gly could then be due to decreased Glu or glycolysis inhibition because the synthesis of both AAs is closely related to Glu levels: through Asp aminotransferase for Asp and glycolytic 3-phosphoglycerate dehydrogenase for Gly [78,79].

Tumour regression *in vivo* and *in vitro* was associated with the induction of ATF4 and inhibition of the mTORC1 pathway in mouse tumour explants By means of *in vitro* silencing experiments, we have shown that ATF4 inhibited the mTORC1 signalling pathway through Sestrin2 − a well-known ATF4 target gene. The downregulation of mTORC1 in mouse xenografts by the AA-defined diet is noteworthy and resembles rapamycin's well-known role, an mTORC1 inhibitor, as an anti-cancer agent [80,81]. Furthermore, our *in vitro* results notably demonstrated that EAAm sensitises cancer cells to the antiproliferative effects of rapamycin, which is otherwise known to be effective only at high doses [44]. In a clinical setting, this could reduce rapamycin doses with beneficial effects in terms of adverse effects.

Additionally, even if not directly and conclusively, our findings suggest that the effects of AA supplementation were cell-autonomous and cancer-specific: the mixture induced apoptotic cell death specifically in cancer cells but not in normal non-cancerous cells. From a clinical point of view, this would result in a lack of side effects and, hence, is of great translational value.

Although intracellular EAA levels were increased in EAAm-treated cells, the inhibition of mTORC1 *in vivo* and *in vitro* after diet feeding and supplementation excludes the risk that augmented AA levels could provide substrates for increased cancer biomass. In line with mTORC1 downregulation, protein synthesis was reduced in the EAAm-treated cells, making unlikely an increase in cell volume or number. Accordingly, our and others’ data increasingly indicate that an “activated mTORC1” is a prerequisite for cell proliferation, independently from the supply of any metabolic substrate. For example, Ananieva et al. showed that BCATm knock-out mice, which display high levels of circulating BCAAs, have a reduced tumour growth; of note, in their animals, mTORC1 was not upregulated [82]. On the contrary, elevated concentrations of BCAAs have led to increased tumour growth when associated with upregulation of mTORC1 activity [83]. Therefore, these findings suggest that high levels of AAs *per se* are not sufficient for an increase in cancer biomass and that, in order to work as a metabolic fuel for cell proliferation, their supply must be linked to the mTORC1 activation.

It is now becoming apparent that AAs fulfil the need for cancers to increase their biomass beyond their well-known role as undefined substrates for protein synthesis. One such example is glutamine (Gln), which, through glutaminolysis, provides carbon skeletons for the TCA cycle and contributes to the NADPH pool used for fatty acid synthesis [84]. However, recent research indicates that other AAs, albeit consumed at much lower rates than Gln, have a significant role in contributing carbon for cell mass instead [85]. Therefore, given the crucial role AAs play in cancer growth as single instead of bulk molecules, several anti-cancer therapeutic approaches to target specific amino acids have been put forward both *in vitro* and *in vivo* [1,28]. Phenylacetate inhibits cancer cell proliferation by reducing Gln availability in the blood [86], while Bis-2-5-phenylacetamido-1,3,4-thiadiazol-2-ylethyl sulfide (BPTES), an inhibitor of the glutaminase—the rate-limiting step in Gln catabolism—blocked the growth of xenografts [29]. Asparaginase, which reduces plasma asparagine, has also been used to treat childhood acute lymphoblastic leukaemia [87], and arginine deiminase has been shown to have antitumour activity [88].

Notably, the SFA-EAA diet decreased intratumor Glu levels in mice, thus confirming that the mechanism of action underlying the specific AA formulation's anticancer effects is also conserved *in vivo*. However, Asp, Gly and Ala were not decreased in tumour samples, thus suggesting that their shortage is probably unnecessary for activating ER stress/mTORC1 inhibition/apoptosis. This is also suggested by the lack of rescue in cell viability when these AAs are re-supplemented in the EAAm mixture (see Figure 5A). Intratumor concentrations of the three BCAAs were increased in SFA-EAA-fed mice, while in plasma from the same animals only Leu and Val were increased, likewise Thr and Tyr (a different AA profile than the EAAm formula) [36]. Accordingly, plasma preparations from SFA-EAA-fed mice were ineffective on cancer cell viability (Supplementary Fig. 5), confirming further the specificity of our EAA formulation in inducing cancer cell death. However, it should be noted that, although the EAAm activates BCAA catabolism (Figure 8), intratumor aminograms from animals fed with our designer diet showed, besides increased BCAAs, variations in levels of other AAs, such as Tyr and Thr (Supplementary Fig. 4). Therefore, we cannot exclude that modifications in the levels of other AAs, resulting from the catabolism of their dietary supply, could be responsible for the starvation of Glu.

The ability of our defined AA formula to induce, both *in vivo* and *in vitro*, a Glu shortage − associated with ATF4 activation and mTORC1 inhibition − suggests for the first time that the AA-starvation may be a beneficial anticancer approach, similar to the previously proposed Gln/Asn targeted therapies [89,90], to obtain with adequate nutritional strategies.

Less is known about the safety and effectiveness of dietary AA supplements in cancer patients. The evidence that tumours utilize AAs [85,91] and that, in particular, high BCAA levels are associated with tumorigenesis [92] raises obvious concerns in clinical settings. Despite these facts, BCAA supplementation has been shown to improve the survival of patients with hepatocellular carcinoma [[93], [94], [95]]. Our results, in conclusion, strengthen these clinical observations, showing that a given dietary strategy specifically impairs cancer growth by reprogramming the AA metabolic sensors. *In vitro* investigation of the mechanism of action showed that this effect was, in turn, triggered by a switch in cellular substrate supply through activation of the mitochondrial BCAA catabolism, which, in turn, inhibited glycolysis. This latter process mimics a starvation-like response, with a fall of the NEAA intracellular levels; the resulting Glu shortage activates ATF4 and ER stress leading to mTORC1 inhibition and cancer cell death.

## 5 Conclusion

From a clinical point of view, our nutritional approach could be associated with other well-established chemotherapeutic pharmacological agents, such as rapalogs and glycolytic inhibitors; this would allow achieving a combined action with a potential reduction of chemotherapeutic dosage and, therefore, possible toxic side effects.

## Author contributions

M.R. designed and performed all of the molecular biology, metabolic, and oxygen consumption experiments; M.R. and C.R. designed and performed experiments in animals; L.T. performed qRT-PCR analysis; E.N. and M.R. conceived the study and designed experiments; M.R. and E.N. wrote the manuscript with suggestions from all authors, all of whom read and approved the final version. M.R. and E.N. are the guarantors of this work and, as such, had full access to all the data in the study and take responsibility for the integrity of the data and the accuracy of the data analysis.

## Funding

This work was supported by 10.13039/501100004710Fondazione Umberto Veronesi to C.R., Professional Dietetics (Milan, Italy) to E.N. (support to laboratory), and 10.13039/501100002803Cariplo Foundation to E.N. and to A.V. (grant 2016-1006).
