# Supplementary material for: An amino acid-defined diet impairs tumour growth in mice by promoting endoplasmic reticulum stress and mTOR inhibition
Source: Mol Metab. 2022 Mar 30;60:101478. doi: 10.1016/j.molmet.2022.101478 (PMC9014392; doi:10.1016/j.molmet.2022.101478)
Supplement: Multimedia component 1 [file mmc1.docx]

**Supplementary data:**


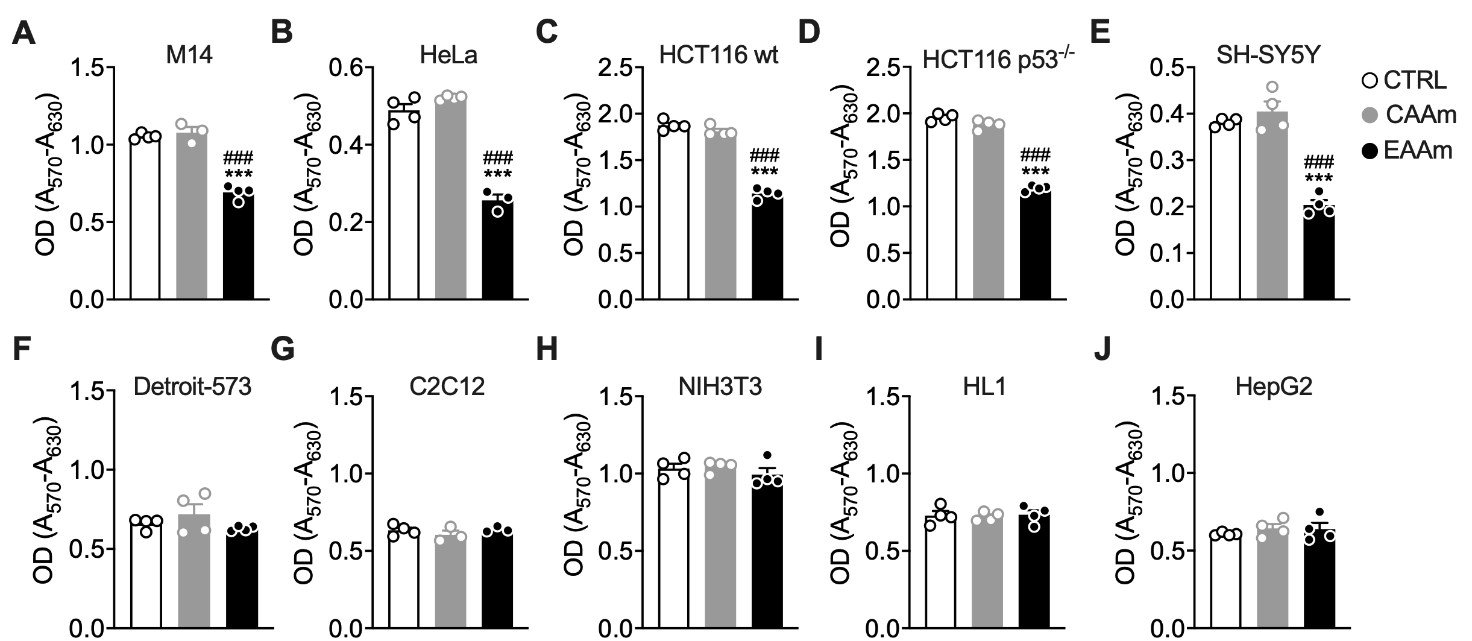


**Supplementary figure 1: Sulforhodamine B assay of viability in both cancer and non-cancer cells**. Cells (n = 4) were seeded and incubated in culture medium (CTRL) or supplemented with EAAm mixture (EAAm) or with a casein-based mixture (CAAm) for 48 h. Afterwards, viability was assessed with a colourimetric Sulforhodamine B (TOX6) assay. Mean (n= 4) ± SEM ****P* < 0.001 vs. CTRL, ^###^*P* < 0.001 vs. CAAm. One-way ANOVA followed by Tukey’s post hoc test.


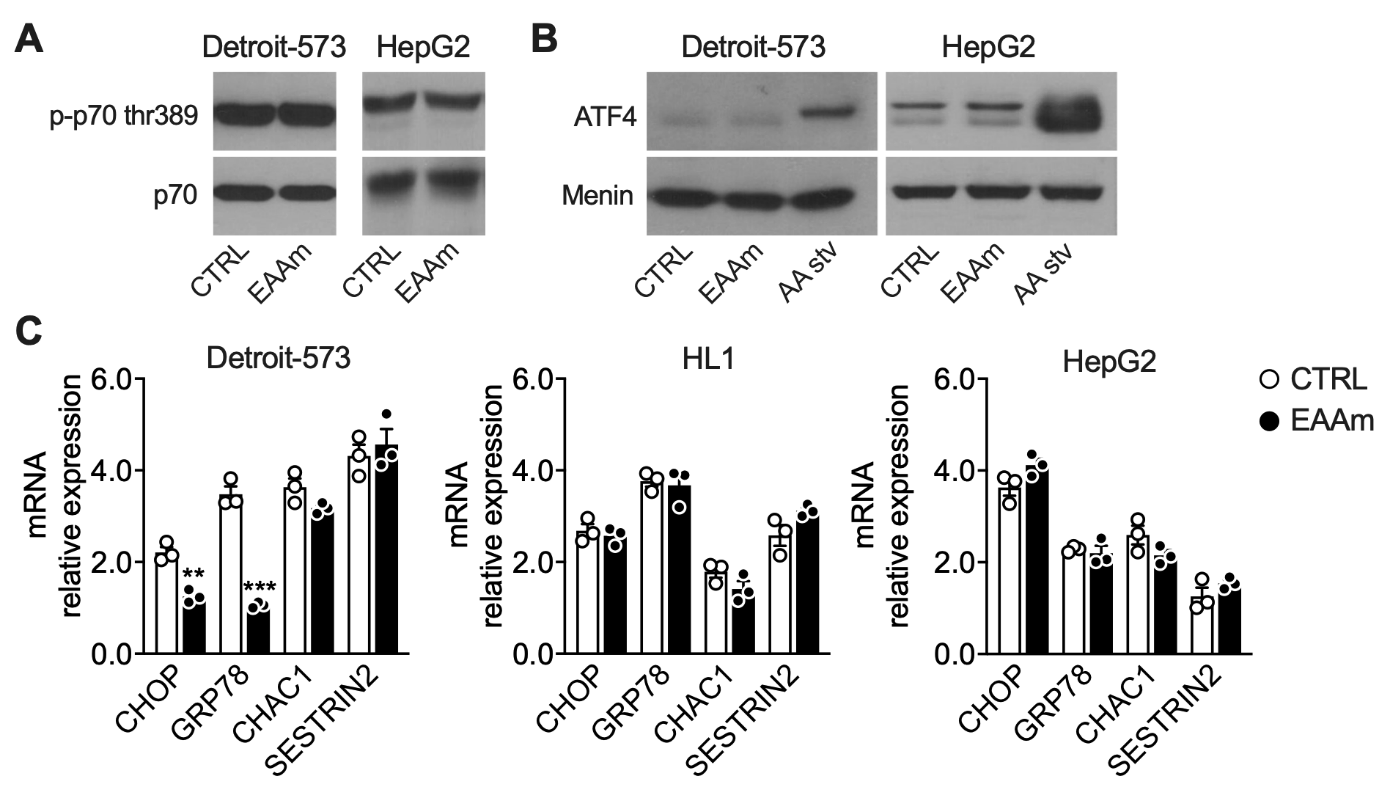


**Supplementary figure 2: EAAm does not affect mTOR, ATF4, and ER stress in non-cancer cell lines**. Western blot of phospho-p70S6K (A) and ATF4 (B), in normal human fibroblasts (Detroit 573) or HepG2 hepatocellular carcinoma cells, which are non-cancerous *in vivo*, incubated in DMEM (CTRL) or EAAm-supplemented medium (EAAm) for 3 hr. In (B), cells were starved of amino acids overnight, as a positive control for the ATF4 induction, and included (AAstv). (C) mRNA expression of ER stress markers in Detroit 573 and HepG2 cells, and HL-1 mouse cardiomyocytes treated as in Figure 6E. Mean (n= 3) ± SEM ***P*<0.005 and ****P* < 0.001 vs CTRL. Unpaired Student’s t-test.

**
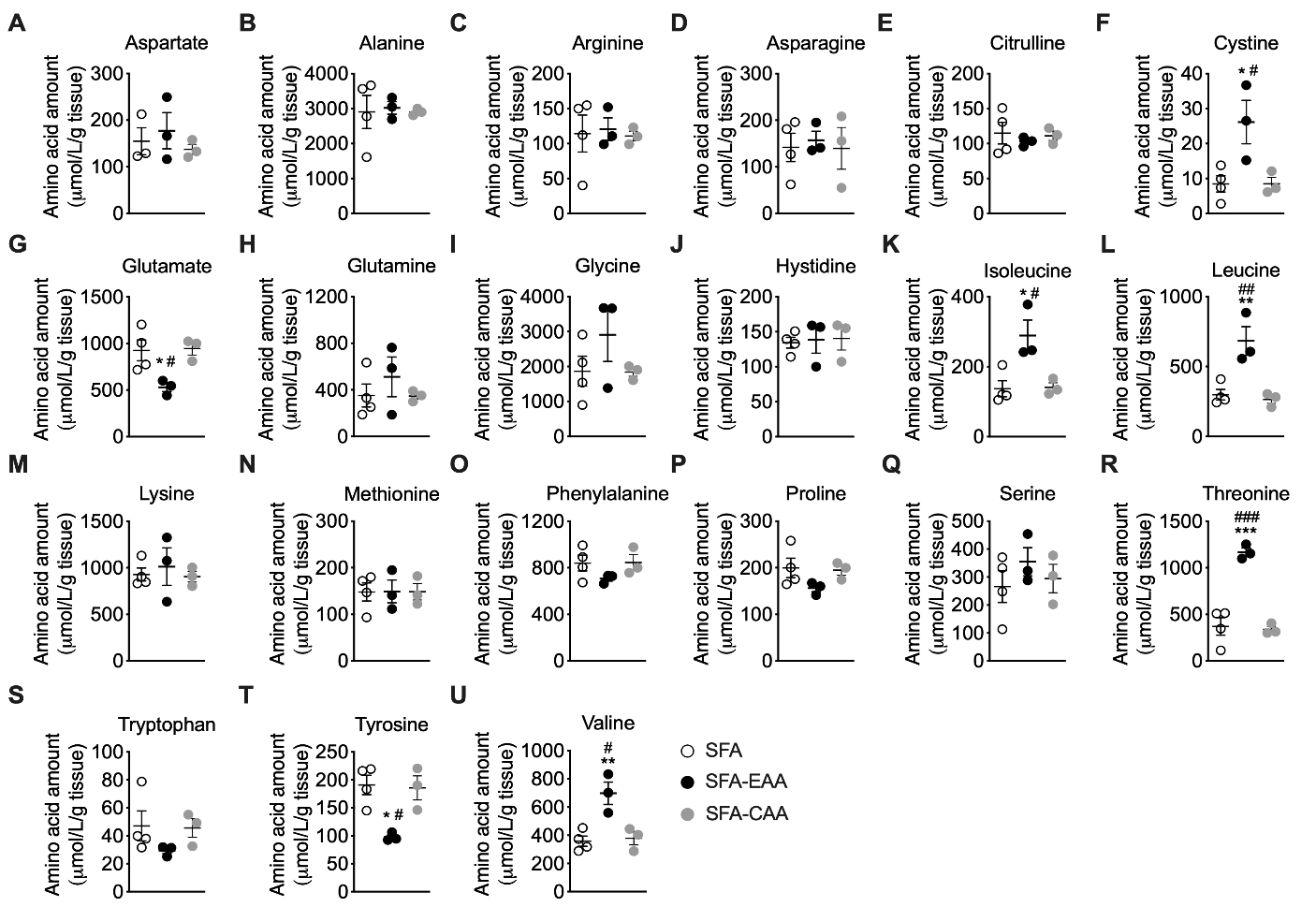
**

**Supplementary figure 3: SFA-EAA diet decreases *in vivo* intratumor Glu levels** (A-U). Aminogram of the twenty amino acids in tumour explants in mice fed with SFA (n=4), SFA-EAA (n=3) and SFA-CAA (n=3). Mice were fed with the designer diets, xenografted and sacrificed as described in “Materials and Methods” (see also Figure 1). Mean ± SEM **P* < 0.05, ***P* < 0.01, and ****P* < 0.001 vs. SFA diet; ^#^*P* < 0.05, and ^###^*P* < 0.001 vs. SFA-CAA diet. one-way ANOVA followed by Tukey’s post hoc test.

**
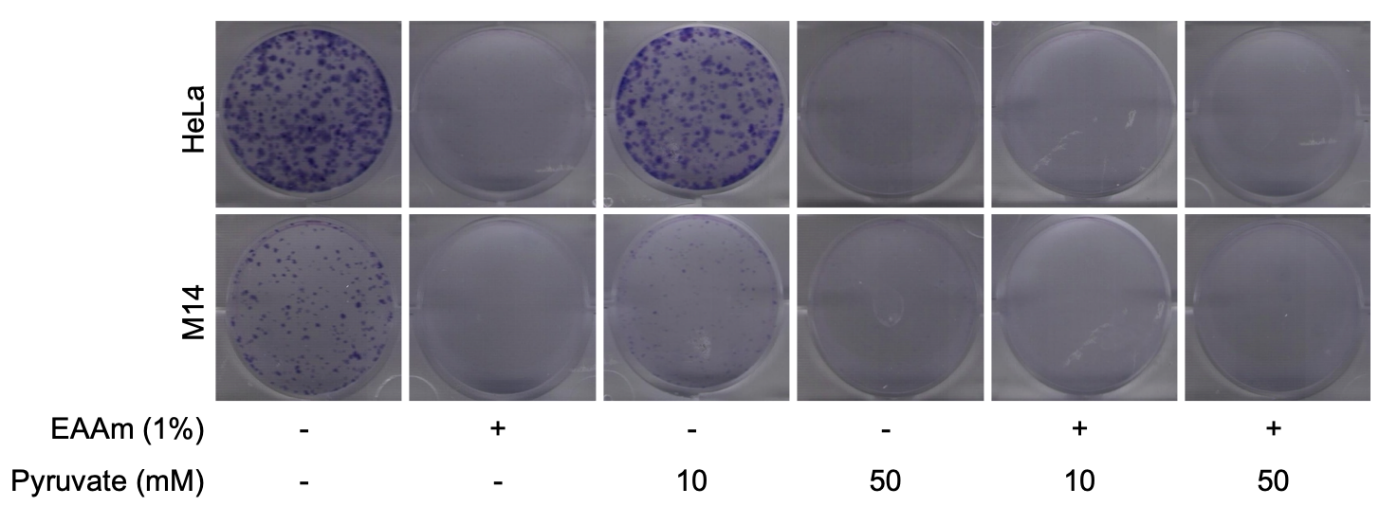
**

**Supplementary figure 4: Pyruvate supplementation does not rescue the impaired clonogenic ability of EAAm-supplemented cancer cells.** Representative data of colony formation assay in M14 and HeLa cells. Twenty-four h after seeding, cells were left untreated, supplemented with 1% EAAm alone or with the indicated concentrations of Pyruvate for ten days.


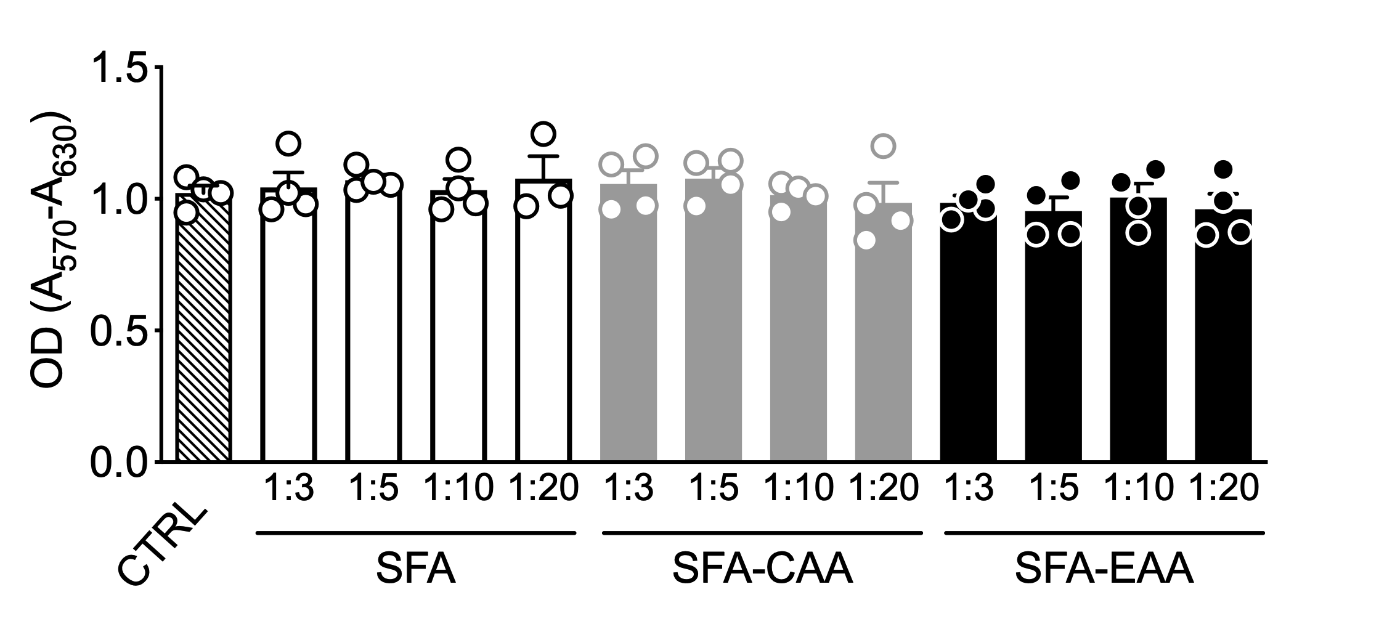


**Supplementary figure 5: Plasma extracts from mice fed with the designer diets and xenografted do not affect cancer cells’ viability.** MTT assay in M14 cells untreated (CTRL) or supplemented with plasma collected from mice fed with the SFA, SFA-CAA or SFA-EAA diet and xenografted (see Figure 1A). Plasma extracts were diluted at the indicated ratios in the culture medium, and cells were incubated for 24 h. Data are Mean (n= 4) ± SEM with two-way ANOVA followed by Tukey’s post hoc test.


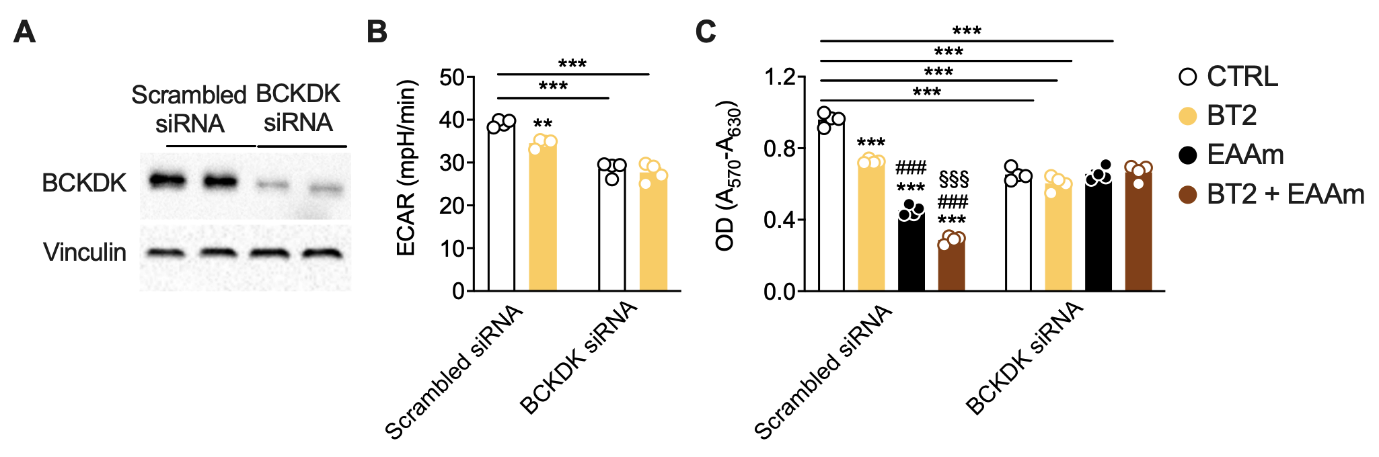


**Supplementary figure 6: BCKDK silencing blocks the effects of BT2 and EAAm on glycolysis and cell viability** (A) Western blot analysis of BCKDK expression in M14 cells transfected for 24 h with non-targeting control siRNA (scrambled) or specific BCKDK siRNA. Duplicate samples are shown. Vinculin is shown as a loading control. (B) ECAR measurement in M14 transfected with scrambled or BCKDK-specific siRNA and left untreated (CTRL) or treated with 100 µM BT2 for 1h (C) MTT assay in M14 transfected with scrambled or siRNA against BCKDK and, after 24h, incubated with culture medium only (CTRL), 100 µM BT2, 1% EAAm or 100 µM BT2 plus 1% EAAm for 24h. Mean (n= 4) ± SEM. ***P* < 0.01 and ****P* < 0.001 vs. CTRL, ^###^*P* < 0.001 vs. BT2, ^§§§^ *P* < 0.001 vs. EAAm. Two-way ANOVA followed by Tukey’s post hoc test.

**Supplementary Table 1**: Macronutrient and micronutrient composition of the mouse diets used in this study.

| Diet | SFA | | SFA-EAA | | SFA-CAA | |
| --- | --- | --- | --- | --- | --- | --- |
| % | gm | Kcal | gm | Kcal | gm | Kcal |
| Protein | 19 | 20 | 19 | 20 | 19 | 20 |
| Carbohydrate | 67 | 70 | 68 | 70 | 68 | 70 |
| Fat | 4 | 10 | 4 | 10 | 4 | 10 |
| Total |  | 100 |  | 100 |  | 100 |
| Kcal/gm | 3.8 |  | 3.8 |  | 3.8 |  |
|  | | | | | | |
| Ingredient | gm | Kcal | gm | Kcal | gm | Kcal |
| Casein | **200** | *800* | **13** | *52* | **13** | *52* |
| L-Alanine | **0** | *0* | **0** | *0* | **5.42** | *22* |
| L-Arginine | **0** | *0* | **0** | *0* | **6.36** | *25* |
| L-Aspartate | **0** | *0* | **0** | *0* | **12.90** | *52* |
| L-Cystine | **3** | *12* | **7.12** | *28* | **1.31** | *5* |
| L-Glutamate | **0** | *0* | **0** | *0* | **40.58** | *162* |
| L-Glycine | **0** | *0* | **0** | *0* | **3.18** | *13* |
| L-Histidine | **0** | *0* | **7.12** | *28* | **4.86** | *19* |
| L-Isoleucine | **0** | *0* | **29.22** | *117* | **8.04** | *32* |
| L-Leucine | **0** | *0* | **58.63** | *235* | **16.83** | *67* |
| L-Lysine | **0** | *0* | **30.34** | *121* | **14.03** | *56* |
| L-Methionine | **0** | *0* | **2.44** | *10* | **5.42** | *22* |
| L-Phenylalanine | **0** | *0* | **4.68** | *19* | **8.98** | *36* |
| L-Proline | **0** | *0* | **0** | *0* | **18.89** | *76* |
| L-Serine | **0** | *0* | **0** | *0* | **10.66** | *43* |
| L-Threonine | **0** | *0* | **16.48** | *66* | **7.67** | *31* |
| L-Tryptophan | **0** | *0* | **0.94** | *4* | **2.24** | *9* |
| L-Tyrosine | **0** | *0* | **1.31** | *5* | **9.72** | *39* |
| L-Valine | **0** | *0* | **29.22** | *117* | **9.91** | *40* |
|  | | | | | | |
| Corn Starch | 452.2 | *1809* | 454.6 | *1818* | 454.6 | *1818* |
| Maltodextrin 10 | 75 | *300* | 75 | *300* | 75 | *300* |
| Sucrose | 172.8 | *691* | 172.8 | *691* | 172.8 | *691* |
| Cellulose, BW200 | 50 | *0* | 50 | *0* | 50 | *0* |
| Soybean Oil | 25 | *225* | 25 | *225* | 25 | *225* |
| Lard | 20 | *180* | 20 | *180* | 20 | *180* |
| Mineral Mix S10026 | 10 | *0* | 10 | *0* | 10 | *0* |
| DiCalcium Phosphate | 13 | *0* | 13 | *0* | 13 | *0* |
| Calcium Carbonate | 5.5 | *0* | 5.5 | *0* | 5.5 | *0* |
| Potassium Citrate, 1 H2O | 16.5 | *0* | 16.5 | *0* | 16.5 | *0* |
| Vitamin Mix V10001 | 10 | *40* | 10 | *40* | 10 | *40* |
| Choline Bitartrate | 2 | *0* | 2 | *0* | 2 | *0* |
| FD&C Red Dye #40 | 0.01 | *0* | 0 | *0* | 0 | *0* |
| FD&C Yellow Dye #5 | 0.04 | *0* | 0 | *0* | 0 | *0* |
| FD&C Blue Dye #1 | 0 | *0* | 0 | *0* | 0.05 | *0* |
| Total | **1055.05** | ***4057*** | **1055.10** | ***4057*** | **1054.90** | ***4057*** |

**Supplementary Table 2**: Amino acid plasma levels of mice fed with the experimental diets. Significative variations in AA levels are shown in bold. Mean (AV) (n= 4) ± SEM. p values < 0.05 were considered significant (one-way ANOVA followed by Tukey’s post hoc test)

|  | **SFA** | | **SFA-CAA** | | *p value* | **SFA-EAA** | | *p value* | *p value* |
| --- | --- | --- | --- | --- | --- | --- | --- | --- | --- |
|  | **AV** | **SEM** | **AV** | **SEM** | vs SFA | **AV** | **SEM** | vs SFA | vs SFA-CAA |
| Aspartic acid | 17.60 | 5.53 | 16.34 | 3.75 | 0.9779 | 9.50 | 1.64 | 0.4397 | 0.5853 |
| Alanine | 253.62 | 69.20 | 332.48 | 63.51 | 0.6541 | 290.47 | 43.89 | 0.8935 | 0.8823 |
| Arginine | 17.35 | 4.99 | 16.51 | 3.92 | 0.9897 | 14.92 | 3.12 | 0.9039 | 0.9631 |
| Asparagine | 21.12 | 7.29 | 25.84 | 3.44 | 0.8775 | 27.83 | 8.54 | 0.7722 | 0.9765 |
| Citrulline | 16.44 | 3.55 | 20.67 | 3.31 | 0.636 | 20.62 | 2.30 | 0.5994 | 0.9999 |
| Cystine | 2.09 | 0.62 | 2.10 | 0.42 | 0.9997 | 0.65 | 0.02 | 0.096 | 0.1202 |
| Glutamate | 68.19 | 14.96 | 68.63 | 6.99 | 0.9998 | 71.28 | 16.95 | 0.987 | 0.9918 |
| Glutamine | 281.95 | 56.73 | 299.59 | 12.71 | 0.9658 | 349.21 | 48.20 | 0.5767 | 0.7661 |
| Glycine | 136.18 | 32.66 | 140.44 | 3.29 | 0.9908 | 146.65 | 12.67 | 0.9372 | 0.9805 |
| Histidine | 22.48 | 3.91 | 24.83 | 4.01 | 0.8964 | 24.35 | 2.83 | 0.9222 | 0.9954 |
| Isoleucine | 21.23 | 3.67 | 18.23 | 2.63 | 0.9945 | 87.55 | 31.82 | 0.0999 | 0.1118 |
| **Leucine** | **35.20** | **7.13** | **23.57** | **0.90** | **0.9768** | **185.48** | **59.76** | **0.0482** | **0.0487** |
| Lysine | 185.04 | 42.72 | 200.59 | 25.26 | 0.9353 | 237.72 | 10.65 | 0.4439 | 0.693 |
| Methionine | 22.78 | 5.76 | 27.00 | 3.30 | 0.7759 | 22.85 | 2.05 | 0.9999 | 0.7824 |
| Phenylalanine | 25.76 | 2.54 | 26.06 | 4.05 | 0.9988 | 27.89 | 5.77 | 0.9339 | 0.9575 |
| Proline | 110.55 | 26.53 | 103.48 | 14.32 | 0.9731 | 73.53 | 18.57 | 0.4544 | 0.6301 |
| Serine | 39.63 | 7.75 | 44.40 | 6.48 | 0.8517 | 40.62 | 2.60 | 0.9918 | 0.9035 |
| **Threonine** | **48.11** | **12.10** | **46.89** | **7.16** | **0.9988** | **167.78** | **25.14** | **0.0031** | **0.0047** |
| Tryptophan | 16.80 | 3.47 | 15.00 | 3.29 | 0.9242 | 14.65 | 2.86 | 0.8779 | 0.997 |
| **Tyrosine** | **37.07** | **6.18** | **40.87** | **9.88** | **0.9043** | **13.51** | **1.49** | **0.0484** | **0.0353** |
| **Valine** | **60.91** | **13.67** | **65.15** | **7.26** | **0.9944** | **187.84** | **42.63** | **0.0281** | **0.0462** |
